# The Redox Activity of Protein Disulfide Isomerase Inhibits ALS Phenotypes in Cellular and Zebrafish Models

**DOI:** 10.1016/j.isci.2020.101097

**Published:** 2020-04-25

**Authors:** Sonam Parakh, Sina Shadfar, Emma R. Perri, Audrey M.G. Ragagnin, Claudia V. Piattoni, Mariela B. Fogolín, Kristy C. Yuan, Hamideh Shahheydari, Emily K. Don, Collen J. Thomas, Yuning Hong, Marcelo A. Comini, Angela S. Laird, Damian M. Spencer, Julie D. Atkin

**Affiliations:** 1 Centre for MND Research, Department of Biomedical Sciences, Faculty of Medicine and Health Sciences, Macquarie University, Sydney, NSW 2109, Australia; 2 Department of Physiology, Anatomy and Microbiology, La Trobe Institute for Molecular Science, La Trobe University, Melbourne, VIC 3086, Australia; 3 Department of Chemistry and Physics, La Trobe Institute for Molecular Science, La Trobe University, Melbourne, VIC 3086, Australia; 4 Department of Biochemistry and Genetics, La Trobe Institute for Molecular Science, La Trobe University, Melbourne, VIC 3086, Australia; 5 Cell Biology Unit, Institut Pasteur de Montevideo, Mataojo 2020, CP 11400 Montevideo, Uruguay; 6 Laboratory Redox Biology of Trypanosomes, Institut Pasteur de Montevideo, Mataojo 2020, CP 11400 Montevideo, Uruguay

**Keywords:** Neurogenetics, Molecular Biology, Neuroscience

## Abstract

Pathological forms of TAR DNA-binding protein 43 (TDP-43) are present in almost all cases of amyotrophic lateral sclerosis (ALS), and 20% of familial ALS cases are due to mutations in superoxide dismutase 1 (SOD1). Redox regulation is critical to maintain cellular homeostasis, although how this relates to ALS is unclear. Here, we demonstrate that the redox function of protein disulfide isomerase (PDI) is protective against protein misfolding, cytoplasmic mislocalization of TDP-43, ER stress, ER-Golgi transport dysfunction, and apoptosis in neuronal cells expressing mutant TDP-43 or SOD1, and motor impairment in zebrafish expressing mutant SOD1. Moreover, previously described PDI mutants present in patients with ALS (D292N, R300H) lack redox activity and were not protective against ALS phenotypes. Hence, these findings implicate the redox activity of PDI centrally in ALS, linking it to multiple cellular processes. They also imply that therapeutics based on PDI's redox activity will be beneficial in ALS.

## Highlights

• The neuronal redox environment is a central event underlying ALS pathogenesis
• PDI's redox activity is protective against mutant TDP-43 and mutant SOD1 in neuronal cells
• PDI's redox activity improves motor impairment in mutant SOD1 zebrafish models
• PDI mutations present in patients with ALS lack redox activity and are not protective

## Graphical Abstract

**Figure:**
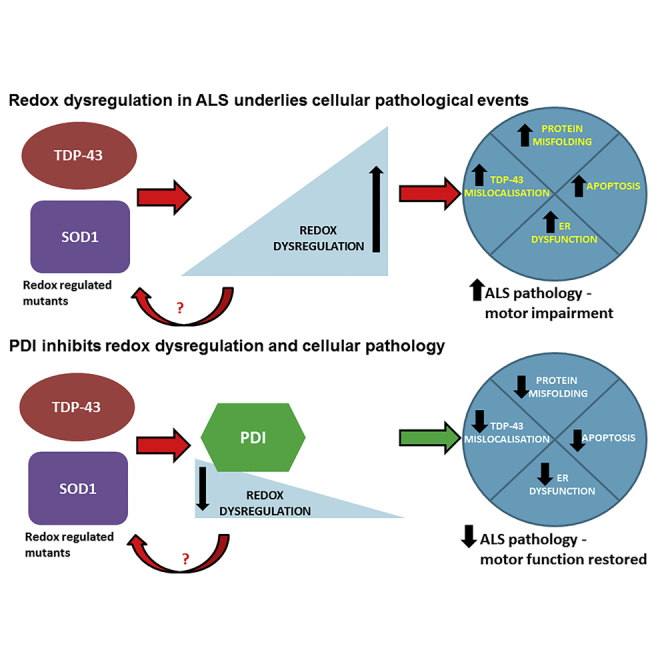

## Introduction

Amyotrophic lateral sclerosis (ALS) is a neurodegenerative disease affecting motor neurons in the brain and spinal cord, leading to progressive loss of motor control (Angelini, 2018), which is related genetically and clinically to frontotemporal dementia (FTD). The majority of ALS cases arise sporadically (sALS), but approximately 10% of cases are familial (fALS) (Leblond et al., 2014), and mutations in the gene encoding cytosolic superoxide dismutase 1 (*SOD1*) cause 20% of fALS cases (Rosen et al., 1993). Mutations in TAR DNA-binding protein (*TARDBP*), encoding TDP-43, represent another 4%–5% of fALS cases (Arai et al., 2006, Neumann et al., 2006). Importantly, aggregated, misfolded TDP-43 mislocalizes from the nucleus to the cytoplasm in almost all cases (97%) and this is recognized to be the major pathological hallmark of ALS (Geser et al., 2008, Neumann et al., 2006, Winton et al., 2008). sALS and fALS are clinically indistinguishable, signifying that an understanding of fALS has broader application to all ALS cases.

Redox homeostasis is now recognized to control a growing number of diverse cellular signaling pathways (Franco and Vargas, 2018). Redox dysregulation is caused by an imbalance between the levels of reactive radicals and antioxidants, and oxidative stress refers to the disruption of redox signaling and control. This changes the cellular redox state, modifies redox proteins, and disrupts redox-regulated mechanisms (Ursini et al., 2016). Although oxidative stress is implicated in ALS, a precise understanding of how redox homeostasis is dysregulated and the resulting cellular consequences are lacking. Nevertheless, redox homeostasis is associated with many pathological mechanisms implicated in ALS, including protein misfolding, endoplasmic reticulum (ER) dysfunction, defects in cellular trafficking, and apoptosis (Atkin et al., 2014, Calabrese et al., 2010, Chang et al., 2013, Cohen et al., 2012, Soo et al., 2015, Walker et al., 2013).

Protein disulfide isomerase (PDI) and ER protein 57 (ERp57) (Matsusaki et al., 2020) are both members of the extended PDI family. Although conventionally regarded as ER proteins, they are also found in multiple other cellular locations, including the cytoplasm (Turano et al., 2002). These unique redox-regulated proteins possess general chaperone activity and are responsible for mediating the formation and rearrangement of disulfide bonds by their oxidoreductase (redox) activity (Matsusaki et al., 2020). PDI consists of four domains, namely a, b, b’, a’. The a and a’ domains harbor the conserved redox active site motif, CGHC, which catalyzes the formation and reduction of disulfide bonds (Kemmink et al., 1996). The b and b’ domains lack redox active cysteine residues but assist in the binding of protein substrates. Ero1p, the major oxidase flavoenzyme at the ER, provides oxidizing equivalents to PDI, whereas the relative levels of oxidized (GSSG) and reduced glutathione (GSH) control PDI and Ero1p activity (Kim et al., 2012, Sevier and Kaiser, 2008). This redox relay system is influenced by the intracellular redox conditions, which if adverse, can lead to changes in protein conformation, misfolding, and degradation (Ellgaard et al., 2017, Feleciano et al., 2016).

Recently, 16 missense mutations, 9 in *P4HB/PDIA1*, the gene encoding PDIA1, and 7 in *PDIA3*, encoding ERp57, were identified in patients with ALS (Gonzalez-Perez et al., 2015, Woehlbier et al., 2016). These mutants are not directly toxic themselves, so it remains unknown how they are involved in neurodegeneration (Woehlbier et al., 2016). PDI associates with misfolded protein inclusions in patients with ALS (Honjo et al., 2011, Parakh et al., 2018), cellular models (Jeon et al., 2014, Farg et al., 2012), and canine degenerative myelopathy (DM) (Chang et al., 2019), and both PDI and ERp57 inhibit the formation of mutant SOD1 inclusions in neuronal cells (Walker et al., 2010, Parakh et al., 2018). Redox-dependent S-nitrosylation of PDI, which inhibits both its chaperone and oxidoreductase activity, is present in patients with ALS (Walker et al., 2010). However, the mechanism by which PDI is associated with ALS is unknown. Furthermore, it is unknown if PDI is protective against pathological forms of TDP-43 or other phenotypes related to ALS, such as TDP-43 mislocalization to the cytoplasm or cellular transport defects. Moreover, a protective role for PDI *in vivo* against misfolded proteins linked to ALS has not yet been demonstrated.

As ALS is a protein misfolding disorder, we predicted that the chaperone activity of PDI would be protective against ALS phenotypes. However, surprisingly, we found that the redox function of PDI was protective against a broad range of events linked to ALS; protein misfolding, mislocalization of TDP-43 to the cytoplasm, ER stress, inhibition of ER-Golgi transport, and apoptosis; in neuronal cells expressing pathological forms of TDP-43 or SOD1. This was confirmed by the finding that PDI ALS mutants (D292N and R300H) lack redox activity and were not protective against mutant TDP-43 or mutant SOD1, implying that in ALS, they lack this normal safeguarding mechanism against aggregation-prone proteins. Similarly, the redox activity of PDI, but not its chaperone function, improved motor phenotype in zebrafish models expressing mutant SOD1. Hence, these findings reveal that the redox activity of PDI regulates multiple cellular processes in ALS. This implicates redox homeostasis as a central mechanism controlling ALS relevant phenotypes, placing it to on a much broader context than previously recognized. These results also predict that therapeutics based on the redox activity of PDI, and not its chaperone function, will be useful in ALS.

## Results

### The Oxidoreductase Activity of PDI Is Protective against Inclusion Formation, Protein Unfolding Induced by Mutant SOD1 and Mutant TDP-43, and TDP-43 Mislocalization into the Cytoplasm

#### Quantification of the Intracellular Redox Environment in Neuro-2a Cells

We initially examined the intracellular redox status of Neuro-2a cells expressing PDI with compounds that modulate redox homeostasis. First, we created a redox inactive mutant of PDI tagged with V5, whereby all four active site cysteine residues were mutated to serine (C53S, C56S, C397S, and C400S, termed 'PDI-QUAD'). We confirmed that the mutations in PDI-QUAD did not affect its subcellular localization in Neuro-2a cells compared with wildtype PDI (PDI-WT); both proteins were ER-localized and non-ER localized to a similar degree (Figure S1A). Second, we obtained similar previously described V5-tagged constructs encoding ALS-associated PDI mutants D292N and R300H (Woehlbier et al., 2016). Third, we modulated the redox environment pharmacologically. BMC ((±)-trans-1,2-Bis (2-mercaptoacetamido) cyclohexane) is a 262 Da synthetic dithiol with a redox potential within physiological values (−240 mV), where the pKa of the first thiol is similar to that of PDI. Hence, BMC is able to mimic the redox activity of PDI (Woycechowsky et al., 1999). Lastly, we used buthionine sulfoximine (BSO) to inhibit glutathione synthesis (Spitz et al., 1995, Hamilos and Wedner, 1985) and thus impede the redox function of PDI. Glutathione modulates the cellular redox environment that maintains PDI in an active form for the oxidation of client proteins (Chakravarthi et al., 2006), and in the presence of glutathione, PDI accelerates the oxidation of disulfide bonds (Darby et al., 1994).

**Figure 1:**
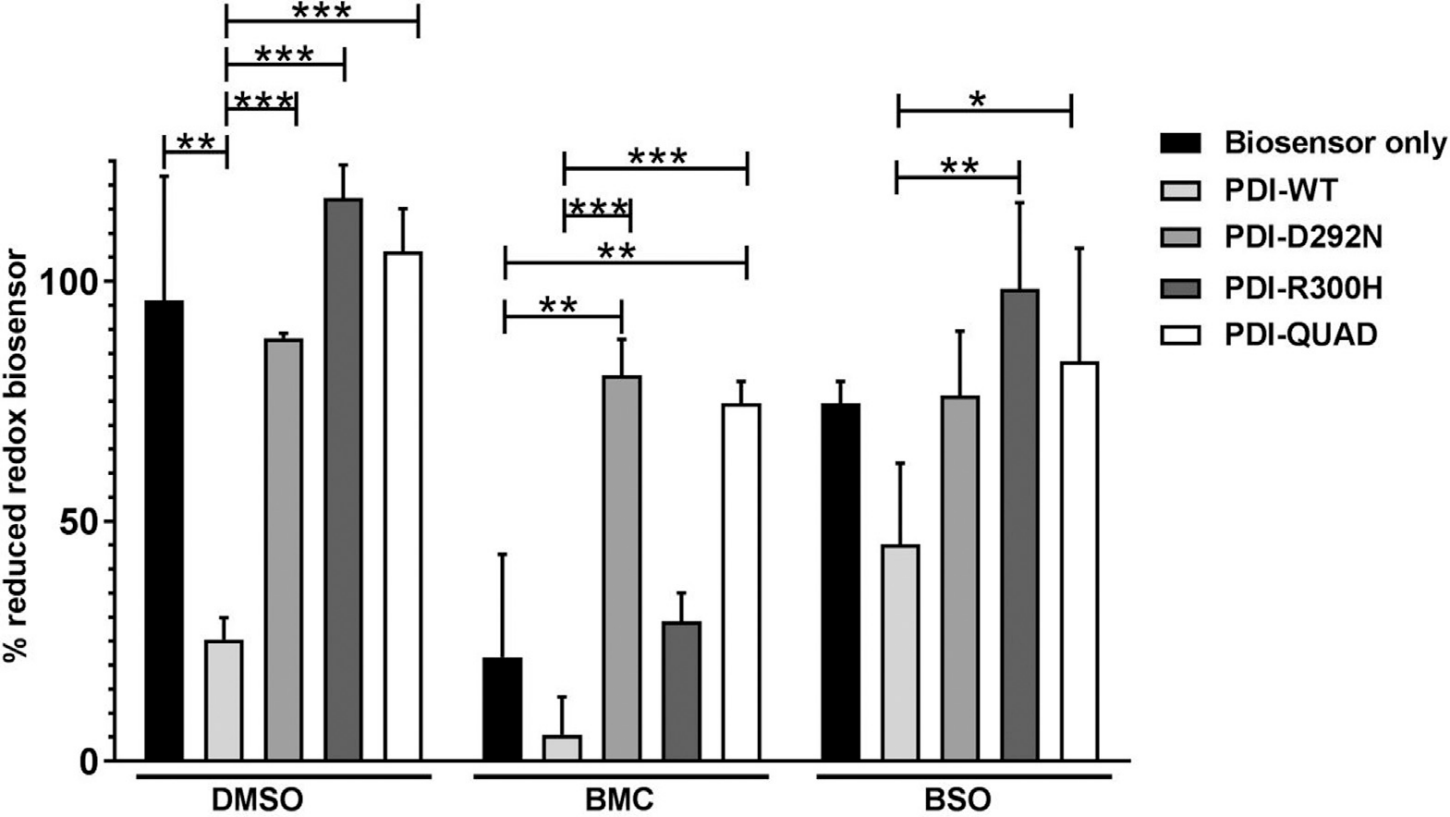
Quantification of the Intracellular Redox Environment in Neuro-2a Cells Reduction of biosensor in Neuro-2a cells transiently transfected with the Clover-rxmRuby2 biosensor (RXM) or with different PDI proteins: PDI-WT, PDI-QUAD PDI-D292N, or PDI-R300H. Statistical difference is shown for biosensor alone expressing cells versus cells expressing different PDI proteins (∗∗p < 0.01 and ∗∗∗p < 0.001) and for cells expressing PDI-WT versus the ALS-associated PDI mutants (∗p < 0.05, ∗∗p < 0.01 and ∗∗∗p < 0.001).

Next, we examined the redox activity of these treatments. For this purpose, we used a genetically encoded redox biosensor, based on the red-shifted mRuby2 fluorescent protein-Clover-rxmRuby2 (Piattoni et al., 2019). This biosensor is expressed in the cytosol, where it provides an overall measurement of the proteins redox state in equilibrium with the GSH/GSSG pool. Neuro-2a cells transiently expressing the redox biosensor alone, and PDI-WT, PDI-D292N, PDI-R300H or PDI-QUAD, treated with BMC, BSO, or dimethyl sulfoxide (DMSO) as vehicle control, were analyzed by flow cytometry (Figure S2A), and the results were plotted as the level (expressed as percentage) of biosensor reduction. Expression of PDI-WT in the presence of DMSO resulted in increased oxidation of the biosensor (25% reduced biosensor) compared with cells expressing the biosensor alone (96% reduced biosensor; p < 0.001, Figure 1), thus confirming PDI's redox activity. However, the redox inactive PDI mutant (QUAD) did not alter the intracellular redox balance, as indicated by 88% reduction of the biosensor. Similarly, expression of PDI mutants PDI-D292N and PDI-R300H had no impact on the redox state of the biosensor (117% and 106% respectively, biosensor reduction). These results therefore demonstrate that under normal conditions, PDI QUAD and the two mutants displayed lower oxidoreductase activity compared with PDI-WT.

Treatment with BMC induced significant intracellular oxidation in cells expressing the redox biosensor alone (22% biosensor reduction) or in those co-expressing PDI-WT (8% reduced biosensor) or PDI-R300H (29% reduced biosensor). In contrast, cells expressing D292N (80% reduced biosensor) or PDI-QUAD (74% reduced biosensor) proved refractory to the effect of BMC (p < 0.01 versus DMSO, p < 0.001 versus PDI-WT).

Compared with the cells expressing biosensor alone (where 96% of reduced biosensor was detected) exposure to BSO induced intracellular oxidation in untransfected cells, as expected for an agent depleting free GSH, resulting in 74% reduced biosensor. In line with the GSH-dependent activity of PDI, the overall intracellular redox state of cells expressing PDI-WT was less oxidative for cultures exposed to BSO (45% biosensor reduction) than for non-BSO-treated cultures (25% biosensor reduction). Cells expressing the different PDI mutants that were treated with BSO displayed intracellular redox milieu (76%, 98%, and 83% biosensor reduction for PDI-D292N, R300H, and QUAD respectively) comparable with that of non-PDI transfected cells (74% biosensor reduction) and far more reducing than cells expressing PDI-WT (45% biosensor reduction). Thus, similar to the data obtained for the full panel of untreated samples, this result supports the redox-silent nature of the mutants PDI-D292N, R300H, and QUAD.

#### The Oxidoreductase Activity of PDI Is Protective against Inclusion Formation and Protein Unfolding Induced by Mutant TDP-43

**Figure 2:**
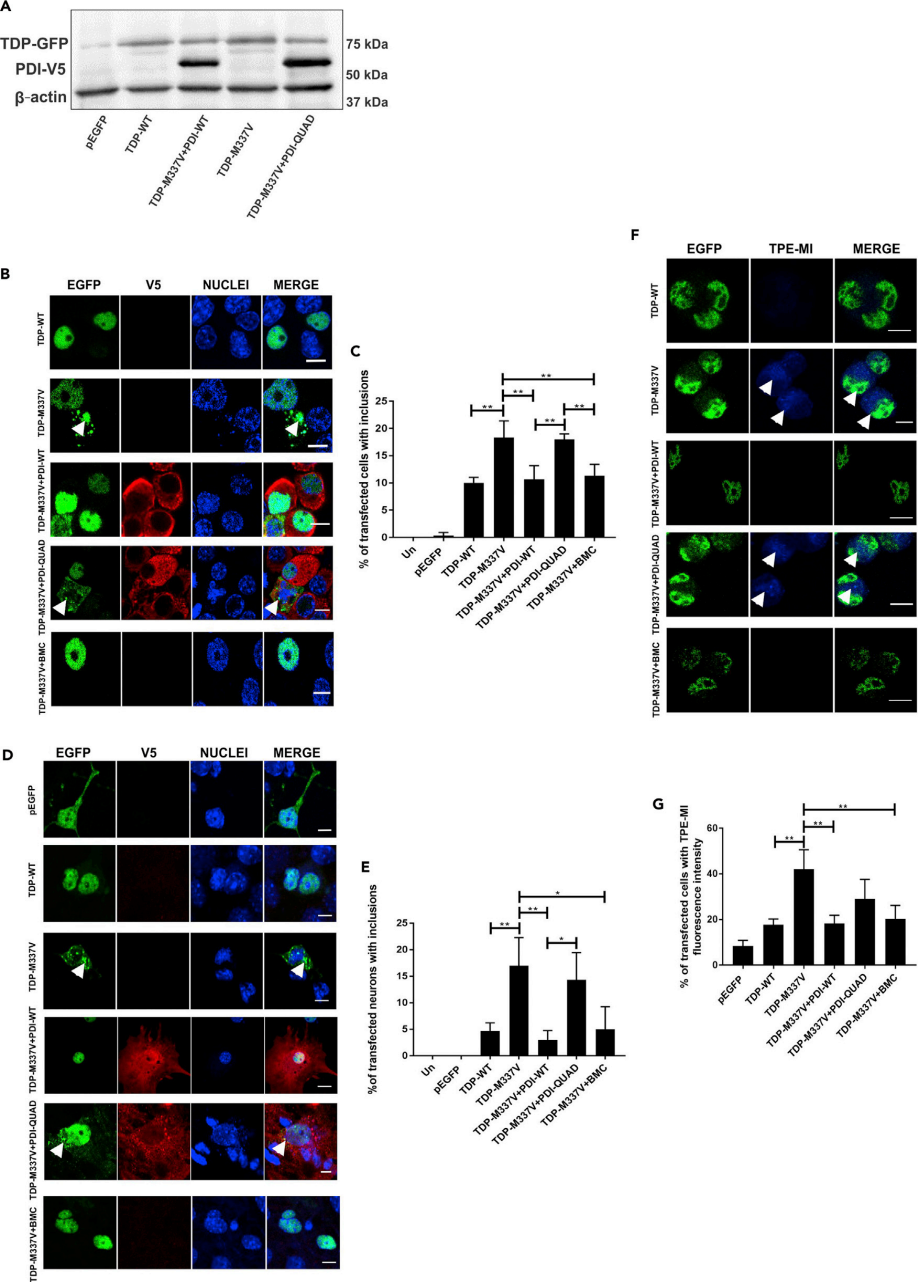
The Oxidoreductase Activity of PDI Is Protective against Inclusion Formation and Protein Unfolding in Mutant TDP-43 Expressing Cells. (A) Immunoblotting was performed to confirm that similar transfection efficiencies were present and that co-expression of PDI-WT or PDI-QUAD did not alter the expression of TDP-43 EGFP. An anti-TDP-43 antibody was used to detect the presence of wild-type TDP-43 (TDP-WT) or mutant TDP-43^M337V^ (TDP-M337V), in cells co-expressing either empty vector pcDNA3.1 or PDI-V5 (WT or QUAD), β-actin was used as a loading control. (B) Immunofluorescence detection of EGFP in cells expressing EGFP-tagged TDP-WT (row 1), TDP-M337V with empty vector alone (row 2) or co-expressing PDI-WT or PDI-QUAD, or administered with BMC (rows 3, 4, 5). (C) Significantly fewer cells formed inclusions when PDI-WT was co-expressed with TDP-M337V or treated with BMC (∗∗p < 0.01). Significant differences were observed between TDP-M337V cells co-expressing PDI-WT or PDI-QUAD, and TDP-M337V cells co-expressing PDI-QUAD or BMC (∗∗p < 0.01). (D) Neurons expressing EGFP only (row 1), TDP-WT (row 2), TDP-M337V alone (row 3), or co-expressing PDI-WT or PDI-QUAD, or BMC-treated (rows 4, 5, 6). (E) Significantly fewer cells formed inclusions when PDI-WT was co-expressed with TDP-M337V (∗∗p < 0.01) and treated with BMC (∗p < 0.05). Significant differences were observed between TDP-M337V cells co-expressing PDI-WT or PDI-QUAD (∗p < 0.05). (F) TPE-MI fluorescence in Neuro-2a cells expressing TDP-WT (row 1), TDP-M337V with empty vector alone (row 2), or co-expressing PDI-WT or PDI-QUAD, or treated with BMC (rows 3, 4, 5), arrows represent TPE-MI fluorescence. (G) Significantly fewer cells displayed TPE-MI fluorescence (representing the cellular load of unfolded proteins, blue) when PDI-WT was co-expressed with TDP-M337V or cells were treated with BMC (∗∗p < 0.01) compared to controls. Scale bars: 8 μm in (B), 5 μm in (D), 12 μm in (F).

We next examined whether PDI is protective against pathological forms of TDP-43. This was investigated in Neuro-2a cells co-expressing enhanced green fluorescent protein (EGFP)-tagged WT or an ALS-associated mutant, TDP-43^M337V^, with V5-tagged PDI-WT or QUAD at 72 h post transfection. Immunoblotting of transfected cell lysates using anti-V5 and anti-TDP-43 antibodies confirmed that the expressed TDP-43 proteins were of the expected size and that PDI did not significantly alter the expression levels of WT or mutant TDP-43 (Figure 2A). Furthermore, quantification revealed that over 99% of cells expressing TDP-43 were also co-expressing PDI (Figure S3A). Hence, it was assumed that detection of TDP-43 expression reflected co-expression of both TDP-43 and PDI in the same cell.

Inclusion formation was examined by fluorescent microscopy following immunocytochemistry using anti-V5 antibodies (Figure 2B). Inclusions were rare in untransfected cells or those expressing EGFP only (<1%), and they were present in only 10% of TDP-WT-expressing cells. As expected, significantly more inclusions were formed (18%, p < 0.01) in cells expressing mutant TDP-43^M337V^, but this proportion was reduced when PDI-WT was co-expressed (11%, p < 0.01, Figure 2C) compared with those expressing EGFP only, unlike PDI-QUAD (18%) where no difference was detected. Similarly, significantly fewer cells treated with BMC (25 μM, 4 h post transfection) formed inclusions (11%, p < 0.01) compared with those treated with DMSO. Hence, these results demonstrate that (i) PDI is protective against the formation of mutant TDP-43 inclusions, and (ii) the oxidoreductase activity of PDI is required for this activity.

To validate these results, mouse cortical primary neurons at embryonic day 16–18 were co-transfected with TDP-43-EGFP and PDI-V5 as above (Figure 2C). Inclusions were absent in control neurons (untransfected and pEGFP), rare in TDP-WT (5%) cells, but ∼3-fold more were present (17%, p < 0.01, Figure 2D) in mutant TDP-43^M337V^-expressing neurons. However, this proportion was significantly reduced when PDI-WT was co-expressed with TDP-43^M337V^ (3%, p < 0.01), unlike PDI-QUAD (14%, Figure 2E), or when cells were treated with BMC (5%, p < 0.05). Hence, these data confirm the results obtained in cell lines that the oxidoreductase activity of PDI is protective against mutant TDP-43 inclusion formation.

We next examined protein unfolding using tetraphenylethene maleimide (TPE-MI) dye, which fluoresces when free cysteine thiols normally buried in the core of globular proteins become exposed during protein unfolding (Chen et al., 2017). Few Neuro-2a cells expressing EGFP alone (8%) or TDP-WT (18%) displayed TPE-MI fluorescence, but significantly more cells expressing mutant TDP-43^M337V^ were fluorescent, as expected (42%, p < 0.01, Figure 2F). However, when PDI-WT was co-expressed with mutant TDP-43, or cells were treated with BMC (18% and 20%, p < 0.01), this proportion was significantly decreased, unlike when PDI-QUAD was co-expressed with mutant TDP-43 (29%, Figure 2G). Hence, these data suggest that the oxidoreductase property of PDI reduces the load of unfolded proteins in cells expressing mutant TDP-43.

#### The Oxidoreductase Activity of PDI Is Protective against Cytoplasmic Mislocalization of Mutant TDP-43

**Figure 3:**
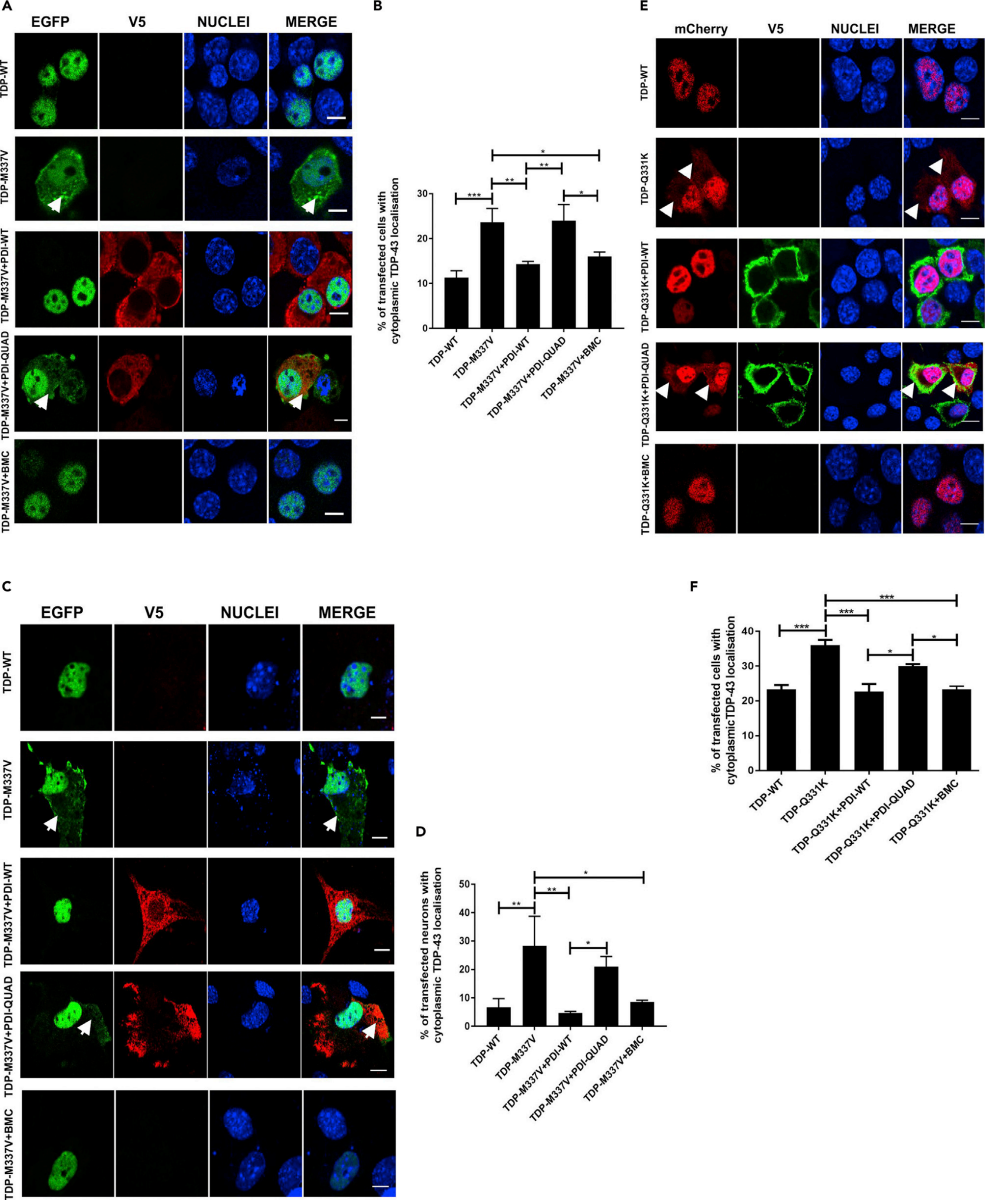
The Oxidoreductase Activity of PDI Is Protective against TDP-43 Mislocalization to the Cytoplasm (A) Neuro-2a cells expressing EGFP-tagged TDP-WT (row 1), TDP-M337V with empty vector (row 2), co-expressing PDI-WT or PDI-QUAD, or treated with BMC (rows 3, 4, 5), arrows repesent mislocalised TDP-43. (B) Expression of PDI-WT (∗∗p < 0.01) or administration of BMC (∗p < 0.05) to mutant TDP-M337V-expressing cells significantly reduced the proportion of cells displaying cytoplasmic TDP-43. A significant difference in cells expressing cytoplasmic TDP-43 was observed between PDI-WT and PDI-QUAD (∗∗p < 0.01), and between PDI-QUAD and BMC (∗p < 0.05). (C) Primary neurons expressing TDP-WT (row 1), TDP-M337V with empty vector (row 2), co-expressing PDI-WT or PDI-QUAD, or treated with BMC (rows 3, 4, 5), arrows repesent mislocalised TDP-43. (D) Over-expression of PDI-WT (∗∗p < 0.01) or BMC treatment (∗p < 0.05) in mutant TDP-M337V-expressing cells significantly reduced the proportion of cells displaying cytoplasmic TDP-43. There was also a significant difference between TDP-M337V cells co-expressing PDI-WT and those co-expressing PDI-QUAD (∗p < 0.05). (E) Neuro-2a cells expressing mCherry-tagged TDP-WT (row 1), TDP-Q331K (row 2), or co-expressing PDI-WT or PDI-QUAD, or treated with BMC (rows 3, 4, 5), arrows repesent mislocalised TDP-43 (F) Expression of PDI-WT or treatment with BMC (∗∗∗p < 0.001) in mutant TDP-Q331K-expressing cells significantly reduced the proportion of cells displaying cytoplasmic TDP-43, compared with cells expressing empty vector only. A significant difference was observed between PDI-WT and PDI-QUAD, and between PDI-QUAD and BMC treated cells (∗p < 0.05). Scale bars: 10 μm in (A) and (E), 5 μm in (C).

Mislocalization of TDP-43 from the nucleus to the cytoplasm is a characteristic pathological hallmark of ALS (Winton et al., 2008). Hence, we next examined whether PDI is protective against the cytoplasmic mislocalization of mutant TDP-43, and if this is mediated by the oxidoreductase activity of PDI, using fluorescent microscopy. Cytoplasmic TDP-43 was detected in only 11% of TDP-WT-expressing Neuro-2a cells, but 2-fold more cells with cytoplasmic expression were present in TDP-43^M337V^ populations (24%, p < 0.001). Co-expression of PDI-WT (14%, p < 0.01) or treatment with BMC (16%, p < 0.05, Figure 3A) resulted in significantly fewer cells with cytoplasmic mutant TDP-43^M337V^, unlike those cells co-expressing PDI-QUAD (24%, Figure 3B), where no significant difference was detected.

To validate these results (Figure 3B), mouse cortical neurons expressing TDP-43-EGFP and PDI-V5 were examined for TDP-43 cytoplasmic localization (Figure 3C). Few primary neurons expressed TDP-WT in the cytoplasm (7%), but 4-fold more neurons (28%, p < 0.01) expressed cytoplasmic mutant TDP-43^M337V^ (with empty vector), as expected. However, co-expression of PDI-WT (5%, p < 0.01) or treatment with BMC (8%, p < 0.05, Figure 3D) resulted in significantly fewer primary neurons expressing cytoplasmic mutant TDP-43, unlike PDI-QUAD where no difference was detected (21%). Hence, these data confirm that the oxidoreductase activity of PDI is protective against TDP-43 mislocalization to the cytoplasm.

As TDP-43 mislocalization to the cytoplasm is central to ALS, additional experiments were performed to confirm these observations. Sporadic ALS TDP-43 mutant Q331K was also examined, bearing a different tag, mCherry, as previously described (Walker et al., 2013), to also confirm that the above results were not restricted to EGFP tagged TDP-43. mCherry-tagged TDP-WT was expressed in the cytoplasm in 23% of cells (Winton et al., 2008), but significantly more was present in TDP-43^Q331K^ cells (36%, p < 0.001, Figure 3E). However, co-expression of PDI-WT or treatment with BMC (23%, p < 0.001) resulted in significantly fewer cells with cytoplasmic mutant TDP-43^Q331K^, unlike cells co-expressing PDI-QUAD where no difference was detected (30%, Figure 3F). Therefore, these data confirm that the oxidoreductase activity of PDI is protective against the mislocalization of mutant TDP-43 in Neuro-2a cells. Hence, together these results show that PDI is protective against typical pathological features of TDP-43 in ALS and that this is mediated by its redox activity.

#### The Oxidoreductase Activity of PDI Is Protective against Inclusion Formation and Protein Unfolding Induced by Mutant SOD1

**Figure 4:**
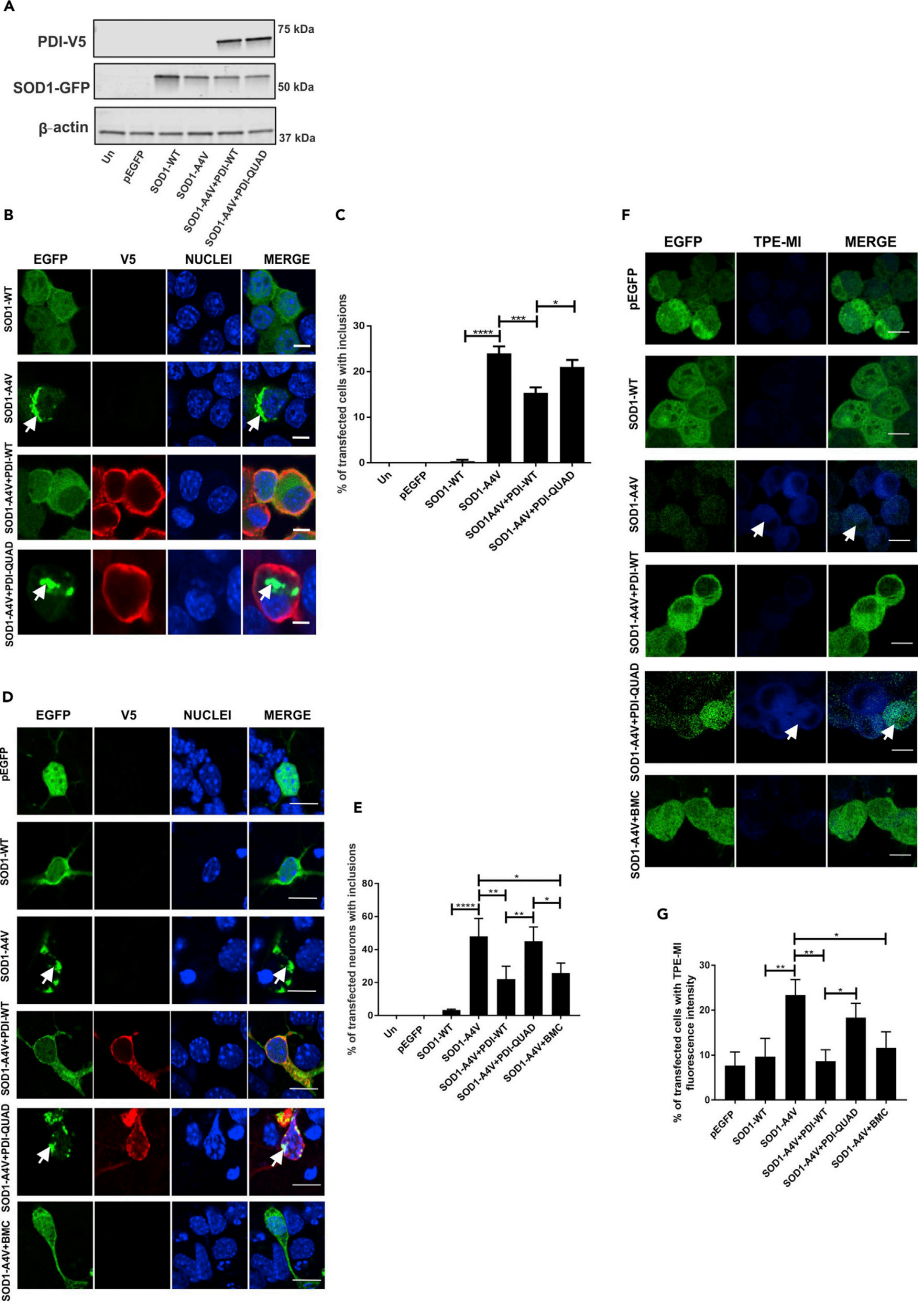
The Oxidoreductase Activity of PDI Is Protective against Inclusion Formation and Protein Unfolding in Mutant SOD1 expressing cells Immunoblotting was performed to confirm that similar transfection efficiencies were present and that co-expression of PDI-WT or PDI-QUAD did not alter the expression of SOD1. An anti-GFP antibody was used to detect SOD1-WT or mutant SOD1^A4V^ (SOD1-A4V), in cells co-expressing empty vector pcDNA3.1 or PDI-V5 (WT or QUAD). β-actin was used as a loading control (bottom panel). (B) Immunofluorescence detection of EGFP in cells expressing SOD1-WT (row 1) or SOD1-A4V (inclusions represented by white arrows, row 2), co-expressed with PDI-WT or PDI-QUAD (rows 3, 4). (C) Significantly fewer cells formed inclusions when PDI-WT was co-expressed with SOD1-A4V (∗∗∗p < 0.001), and significant difference was observed between PDI-WT and PDI-QUAD expressing cells (∗p < 0.05). (D) Immunofluorescence detection of EGFP-positive inclusions present in mouse primary neurons co-expressing EGFP only (row 1), SOD1-WT (row 2) or SOD1-A4V (row 3), with PDI-WT or PDI-QUAD, or treated with BMC (rows 4, 5, 6). (E) Significantly fewer neurons formed inclusions when PDI-WT was co-expressed with SOD1-A4V (∗∗p < 0.01) or treated with BMC (∗p < 0.05). A significant difference was observed between SOD1-WT and mutant SOD1-A4V (∗∗∗∗p<0.0001) cells. n = 35, ANOVA followed by Tukey's post hoc test. (F) TPE-MI fluorescence in Neuro-2a cells expressing pEGFP (row 1), SOD1-WT (row 2), or SOD1-A4V cells (row 3), co-expressing PDI-WT or PDI-QUAD, or treated with BMC (rows 4, 5, 6). (G) Significantly fewer cells displayed TPE-MI fluorescence when PDI-WT was co-expressed with SOD1-A4V or treated with BMC (∗∗p < 0.01 and ∗p < 0.05). Scale bars: 10 μm in (B), 10 μm in (D), 15 μm in (F).

We next examined whether the oxidoreductase property of PDI is protective against mutant SOD1^A4V^ (SOD1-A4V). Neuro-2a cells were co-transfected with EGFP-tagged wild-type SOD1 (SOD1-WT) or SOD1^A4V^, and PDI-WT, PDI-QUAD, or empty vector pcDNA3.1. Similar levels of expression between PDI-WT and PDI-QUAD were observed, and PDI did not alter the expression levels of either WT or mutant SOD1 (Figure 4A). Quantification revealed that over 99% of cells expressing SOD1 were also co-expressing PDI (Figure S4A); hence, it was assumed that detection of SOD1 expression reflected co-expression of both SOD1 and PDI in the same cell.

At 72 h post transfection, following immunochemistry using anti-V5 antibodies, the percentage of cells bearing mutant SOD1 inclusions was quantified microscopically (Figure 4B). Similar to previous observations (Walker et al., 2010, Parakh et al., 2018), inclusions were formed in 24% of cells expressing mutant SOD1^A4V^ but were negligible (<1%) in untransfected cells (Un) and cells expressing EGFP alone or SOD1-WT. Following co-expression of PDI-WT, as previous (Walker et al., 2010) this proportion was significantly reduced (15%, p < 0.001), unlike co-expression of PDI-QUAD (21%, Figure 4C). Hence, these data suggest that the oxidoreductase activity of PDI is required for its protective activity against mutant SOD1 inclusion formation.

To validate these results (Figure 4C), primary neurons were examined for inclusion formation (Figure 4D). As previous (Parakh et al., 2018), 48% (p < 0.0001) of primary neurons co-expressing mutant SOD1^A4V^ with empty vector (pcDNA3.1) formed inclusions. However, this proportion was significantly reduced to 22% (p < 0.01) when PDI-WT was co-expressed with mutant SOD1^A4V^ (Figure 4E), unlike PDI-QUAD (45%) where no difference was detected. Few inclusions were formed in neurons expressing EGFP only or SOD1-WT (3%), as expected. To further validate these results, mutant SOD1-expressing primary neurons were treated with BMC. Significantly fewer neurons contained inclusions compared with cells treated with vehicle only (DMSO, 26%, p < 0.05). Taken together, these data confirm that the oxidoreductase activity of PDI confers protection against mutant SOD1 inclusion formation.

Next protein unfolding was examined in cells expressing PDI and SOD1. Few Neuro-2a cells expressing EGFP alone (8%) or SOD1-WT (10%) displayed TPE-MI fluorescence, whereas 23% of cells expressing mutant SOD1^A4V^ were fluorescent (p < 0.01, Figure 4F). Consistent with previous observations, TPE-MI did not label mutant SOD1^A4V^ inclusions (Chen et al., 2017). Co-expression of PDI-WT (9%, p < 0.01) or treatment with BMC (12%, p < 0.05) significantly reduced the proportion of SOD1^A4V^ cells with TPE-MI fluorescence, whereas PDI-QUAD had no effect (18%, Figure 4G). These data therefore reveal that the oxidoreductase property of PDI reduces the load of unfolded proteins in cells expressing mutant SOD1^A4V^. Hence, overall, these results demonstrate that the redox activity of PDI is protective against inclusion formation, cytoplasmic mislocalization and protein unfolding, in cells expressing pathological forms of TDP-43 and SOD1.

### The Oxidoreductase Activity of PDI Is Protective against ER Defects Induced by Pathological Forms of TDP-43 and SOD1

Both mutant TDP-43 and mutant SOD1 induce ER stress, which can be detected by nuclear immunoreactivity to XBP-1 and CHOP (Walker et al., 2010, Walker et al., 2013), and we have previously demonstrated that these are highly sensitive methods to detect UPR activation that do not depend on transfection efficiency (Walker et al., 2010, Walker et al., 2013, Soo et al., 2015, Parakh et al., 2018). The presence of nuclear XBP-1 detects activation of IRE-1, and the extent of IRE1 signaling matches the magnitude of the stress (Pincus et al., 2010). CHOP, activated by the PERK and ATF6 pathways, mediates the transition of the UPR into its apoptotic phase (Szegezdi et al., 2006).

#### The Oxidoreductase Activity of PDI Is Protective against ER Stress Induced by Mutant TDP-43

**Figure 5:**
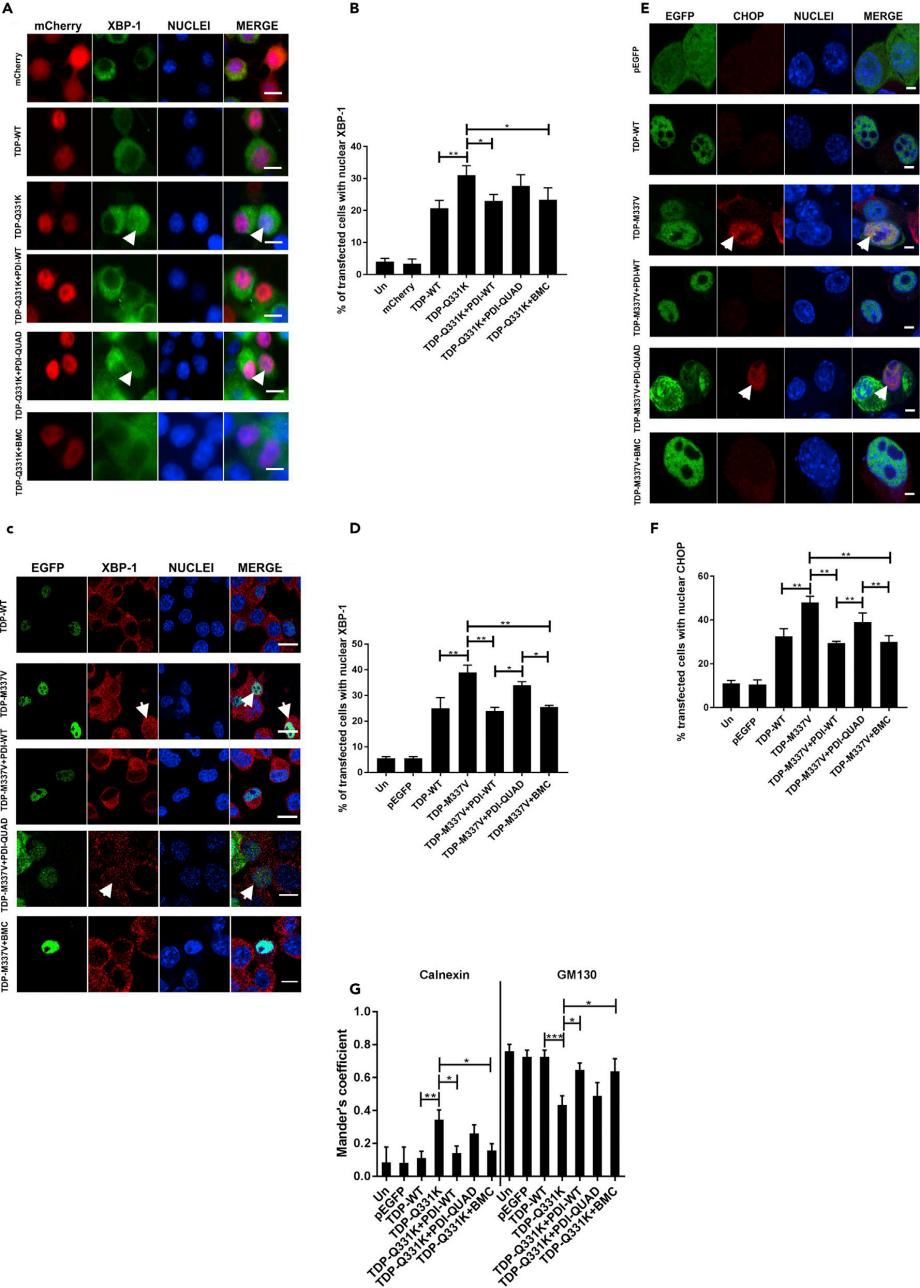
The Oxidoreductase Activity of PDI Is Protective against ER Stress and Inhibition of ER-Golgi Transport Induced by Mutant TDP-43 (A) Detection of nuclear immunoreactivity to XBP-1 in cells expressing mCherry-tagged TDP-43. Cells expressing mCherry (row 1), TDP-WT (row 2), or mutant TDP-Q331K (row 3), co-expressing PDI-WT or PDI-QUAD, or treated with BMC (rows 4, 5, 6), arrows representing XBP-1 activation. (B) The proportion of cells expressing nuclear XBP-1 decreased when PDI-WT was co-expressed or treated with BMC (∗p < 0.05), unlike PDI-QUAD. (C) Immunofluorescence detection of nuclear immunoreactivity to XBP-1 in EGFP-tagged TDP-43 cells. Cells expressing TDP-WT (row 1), or TDP-M337V (row 2), co-expressing PDI-WT or PDI-QUAD, or treatment with BMC (rows 3, 4, 5), arrows represent XBP-1 activation. (D) The proportion of cells expressing nuclear XBP-1 decreased when PDI-WT was co-expressed, or BMC was administered to TDP-M337V cells (∗∗p < 0.01). More cells with nuclear XBP-1 were found in populations expressing PDI-QUAD compared with PDI-WT, and PDI-QUAD compared with BMC treatment (∗p < 0.05). (E) Detection of nuclear immunoreactivity to CHOP in EGFP-tagged TDP-43 cells. Cells expressing pEGFP (row 1), TDP-WT (row 2), TDP-M337V (row 3), co-expressing PDI-WT or PDI-QUAD, or treated with BMC (rows 4, 5, 6), arrows represent CHOP activation. (F) The proportion of cells expressing nuclear CHOP was decreased when PDI-WT was co-expressed or BMC was administered to TDP-M337V cells (∗∗p < 0.01). There was a significant difference between TDP-M337V cells co-expressing PDI-WT and PDI-QUAD (∗∗p < 0.01), and TDP-M337V cells co-expressing PDI-QUAD and treated with BMC (∗∗p < 0.01). (G) PDI's oxidoreductase activity rescues inhibition of ER-Golgi transport induced by mutant TDP-43. Quantification of the degree of co-localization of VSVG^ts045^ with the ER and Golgi compartments using Mander's coefficient following immunocytochemistry for calnexin and GM130. Data are presented as mean ± SEM, n = 20. A significant difference was observed (∗p < 0.05) in the co-localization between VSVG^ts045^ and the ER (calnexin) between cells expressing TDP-Q331K with empty vector and those expressing PDI-WT, and also with (∗p < 0.05) BMC-treatment . A significant difference was also observed (∗p < 0.05) in co-localization between VSVG^ts045^ and the Golgi (GM130) between cells expressing TDP-Q331K and PDI-WT and BMC-treated cells. Scale bars: 10 μm in (A) and (C), 4 μm in (E).

Neuro-2a cells were co-transfected as above and immunocytochemistry was performed using anti-XBP-1 or anti-CHOP antibodies, followed by microscopy. Two TDP-43 mutants were examined initially, mCherry-tagged TDP-43^Q331K^ (Figure 5A) and EGFP-tagged TDP-43^M337V^ (Figure 5C), in cells expressing either empty vector, PDI-WT or PDI-QUAD, or those treated with BMC. Nuclear immunoreactivity to XBP-1 was negligible in control cells (untransfected cells, mCherry, and pEGFP-expressing cells [≤6%]) but was present in 21% mCherry-tagged TDP-WT and 25% of GFP-tagged TDP-WT-expressing cells, similar to previous observations (Walker et al., 2013). In contrast, significantly more cells expressing mutants TDP-43^Q331K^ (31%, p < 0.01) or TDP-43^M337V^ (39%, p < 0.01) displayed nuclear XBP-1, indicating activation of ER stress. However, this proportion was significantly lower when PDI-WT was co-expressed with either TDP-43^Q331K^ (23%, p < 0.05) or TDP-43^M337V^ (24%, p < 0.01) or when cells were treated with BMC (23%, p < 0.05, Figure 5B and 25%, p < 0.01, Figure 5D, respectively), whereas PDI-QUAD had no effect in either case (28% and 34%). Hence, the oxidoreductase activity of PDI is protective against nuclear immunoreactivity to XBP-1.

We next confirmed these results by examining CHOP nuclear immunoreactivity in cells expressing EGFP-tagged TDP-43^M337V^ (Figure 5E). Nuclear immunoreactivity was detected in 10%–11% of untransfected or EGFP only cells, and 32% of TDP-WT GFP-expressing cells. More TDP-43^M337V^ cells displayed nuclear CHOP (48%, p < 0.01), indicating activation of pro-apoptotic UPR, but this proportion was significantly reduced by co-expression with PDI-WT (30%) or treatment with BMC (30%, p < 0.01, Figure 5F). In contrast, co-expression of PDI-QUAD (39%) did not affect nuclear CHOP immunoreactivity. Hence, these data confirm that the oxidoreductase activity of PDI is essential for protection against ER stress induced by mutant TDP-43.

#### The Oxidoreductase Activity of PDI Is Protective against Inhibition of ER-Golgi transport Induced by Mutant TDP-43

We also previously demonstrated that mutant SOD1 and mutant TDP-43 inhibit vesicular transport between the ER and Golgi apparatus and that this is associated with ER stress (Soo et al., 2015, Atkin et al., 2014). Hence, next, inhibition of ER-Golgi transport by mutant TDP-43 EGFP was examined using a temperature-sensitive mutant of vesicular stomatitis viral glycoprotein (VSVG^ts045^) tagged with-mCherry (see Transparent Methods). We used VSVG^ts045^ because it is a classical transport marker used to examine trafficking from the ER to Golgi (Presley et al., 1997, Soo et al., 2015). Neuro-2a cells were co-transfected with mCherry-VSVG^ts045^, TDP-WT, or mutant TDP-43^Q331K^ and either empty vector or V5-tagged PDI (WT or QUAD). Alternatively, cells were treated with BMC. Quantification of the localization of VSVG^ts045^ in either the ER or Golgi compartments was performed using Mander's co-efficient, where a value of 0 represents no co-localization and a value of 1 denotes total co-localization due to full overlapping of pixels (Atkin et al., 2014). For untransfected cells (Un), EGFP only or TDP-WT-expressing cells, a minor proportion of VSVG^ts045^ (8%–11%) was retained in the ER and most (72%–75%) was transported to the Golgi apparatus after 30 min. In cells expressing mutant TDP-43^Q331K^, ER-Golgi transport was inhibited (34% versus 43% VSVG^ts045^ in ER versus Golgi, p < 0.01 and p < 0.001, respectively) as previously described (Soo et al., 2015). However, when PDI-WT was co-expressed with mutant TDP-43^Q331K^, transport between the ER-Golgi was restored; less VSVG^ts045^ was present in the ER (14%, p < 0.05) and more was detected in the Golgi compared with mutant TDP-43 transfected with empty vector (64%, p < 0.05, Figure 5G). Similar results were obtained in cells treated with BMC (15% versus 63% VSVG^ts045^ in ER versus Golgi, p < 0.05, Figure S5A). In contrast, co-expression of PDI-QUAD with TDP-43^Q331K^ (26% VSVG^ts045^ in the ER and 50% in the Golgi) did not restore transport. Hence, these data reveal that the oxidoreductase activity of PDI is protective against mutant TDP-43-induced inhibition of ER-Golgi transport.

#### The Oxidoreductase Activity of PDI Is Protective against ER Stress Induced by Mutant SOD1

**Figure 6:**
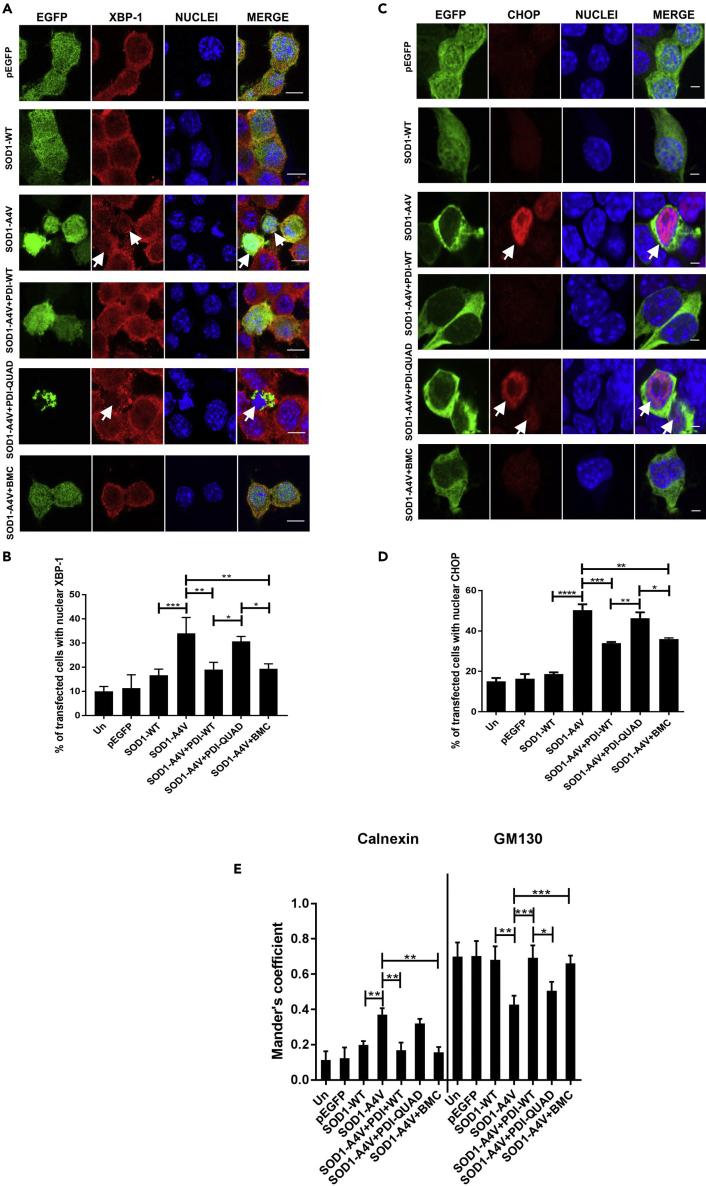
The Oxidoreductase Activity of PDI Is Protective against ER Stress and Inhibition of ER-Golgi Transport Induced by Mutant SOD1 (A) Detection of nuclear immunoreactivity to XBP-1 (second column) in EGFP (row 1), SOD1-WT (row 2) or SOD1-A4V (row 3) cells, co-expressing either PDI-WT or PDI-QUAD, or treated with BMC (rows 4, 5, 6), arrows represent XBP-1 activation. (B) Fewer cells expressing nuclear XBP-1 were present when PDI-WT was co-expressed or cells were treated with BMC (∗∗p < 0.01). There was a significant difference between SOD1-WT and SOD1-A4V cells (∗∗∗p<0.001). Similarly, a significant difference was observed between SOD1-A4V cells co-expressing PDI-WT or PDI-QUAD, and SOD1-A4V cells co-expressing PDI-QUAD or treated with BMC (∗p < 0.05). (C) Immunofluorescence detection of nuclear immunoreactivity to CHOP (second column) in cells expressing EGFP (row 1), SOD1-WT only (row 2), or SOD1-A4V (row 3) with PDI-WT or PDI-QUAD, or treated with BMC (rows 4, 5, 6), arrows represent CHOP activation. (D) The proportion of cells expressing nuclear CHOP was significantly decreased when PDI-WT was co-expressed with SOD1-A4V (∗∗∗p < 0.001) or treated with BMC (∗∗p < 0.01). There was significant difference between SOD1-WT and SOD1-A4V cells (∗∗∗∗p<0.0001). Significant differences were also detected between SOD1-A4V cells co-expressing PDI-WT and PDI-QUAD (∗∗p < 0.01), and SOD1-A4V cells co-expressing PDI-QUAD or treated with BMC (∗p < 0.05). (E) PDI's oxidoreductase activity rescues inhibition of ER-Golgi transport induced by mutant SOD1. Quantification of the degree of co-localization of VSVG^ts045^ with the ER and Golgi compartments using Mander's coefficient following immunocytochemistry for calnexin and GM130. Data are presented as mean ± SEM, n = 20. More co-localization between VSVG^ts045^and the ER (calnexin) was observed (∗∗p < 0.01) in SOD1-A4V cells compared with those co-expressing PDI-WT or treated with BMC. More SOD1-A4V and PDI-WT co-expressing cells, or those treated with BMC, displayed co-localization between VSVG^ts045^ and the Golgi (GM130) (∗∗∗p < 0.001). Similarly, there was a significant difference in Golgi localisation between cells expressing PDI-WT and PDI-QUAD (∗p < 0.05). Scale bars: 10 μm in (A), 4 μm in (C).

Similarly, ER stress induced by mutant SOD1 was next examined using XBP-1 and CHOP nuclear immunoreactivity. As previous, few control cells (10% untransfected cells, 11% EGFP and 17% SOD1-WT-expressing cells) (Parakh et al., 2018) and significantly more mutant SOD1^A4V^ cells without PDI (co-expressing empty vector pcDNA3.1) displayed nuclear XBP-1 immunoreactivity, indicating activation of IRE1 signaling and hence ER stress (34%, p < 0.001, Figure 6A). However, the proportion of mutant SOD1^A4V^ cells was significantly reduced by co-expression of PDI-WT or by treatment with BMC (19%, p < 0.01), but not PDI-QUAD (31%, Figure 6B). Second, ≤ 19% of control cells (untransfected, pEGFP and SOD1-WT-expressing) and significantly more SOD1^A4V^ cells without PDI (50%, p < 0.0001) (Walker et al., 2010) displayed nuclear CHOP immunoreactivity, indicating activation of proapoptotic UPR (Figure 6C). However, treatment with BMC (36%, p < 0.01) or co-expression of PDI-WT (34%, p < 0.001), but not PDI-QUAD (46%, Figure 6D), significantly reduced this proportion. These results therefore reveal that the oxidoreductase activity of PDI is protective against ER stress induced by mutant TDP-43 ^M337V^ and mutant SOD1^A4V^.

#### The Oxidoreductase Activity of PDI Is Protective against Inhibition of ER-Golgi transport Induced by Mutant SOD1

We next examined whether PDI is protective against mutant SOD1-induced ER-Golgi transport dysfunction. Neuro-2a cells were co-transfected with mCherry-tagged VSVG^ts045^ and either EGFP-tagged SOD1-WT or mutant SOD1^A4V^, and empty vector or V5-tagged PDI (WT or QUAD). Alternatively, SOD1 and VSVG^ts045^-expressing cells were also treated with BMC or empty vehicle DMSO (Figure S5B). In untransfected cells (11%), and cells expressing pEGFP (12%) or SOD1-WT (19%), most VSVG^ts045^ was transported to the Golgi apparatus after 30 min at the permissive temperature, and only a minor fraction was retained in the ER. Similar to previous observations (Atkin et al., 2014, Soo et al., 2015), in cells expressing mutant SOD1^A4V^ more VSVG^ts045^ was retained in the ER (37%, p < 0.01) and less was present in the Golgi (42%, p < 0.01) (Figure 6E). However, when PDI-WT was co-expressed with mutant SOD1^A4V^, transport between the ER and Golgi was restored. Similar to control cells, 21% (p < 0.01) VSVG^ts045^ was now retained in the ER and 69% (p < 0.001) was found in the Golgi. Similar results were obtained for cells expressing SOD1^A4V^ treated with BMC, only 16% (p < 0.01) of VSVG^ts045^ was detected in the ER and 66% (p < 0.001) was present in the Golgi. In contrast, when PDI-QUAD was co-expressed with mutant SOD1^A4V^ ER-Golgi transport was not restored; significantly more VSVG^ts045^was retained in the ER (30%) and less was transported to the Golgi (50%) compared with cells expressing empty vector. Therefore, these data reveal that both PDI-WT and BMC, but not PDI-QUAD, restore ER-Golgi transport inhibited by mutant SOD1. Hence, together these results show that the oxidoreductase activity of PDI is protective against inhibition of ER-Golgi transport induced by both mutant SOD1 and mutant TDP-43.

### Both the Oxidoreductase Activity and Chaperone Activity of PDI Are Protective against Apoptotic Cell Death Induced by Mutant TDP-43 and Mutant SOD1

**Figure 7:**
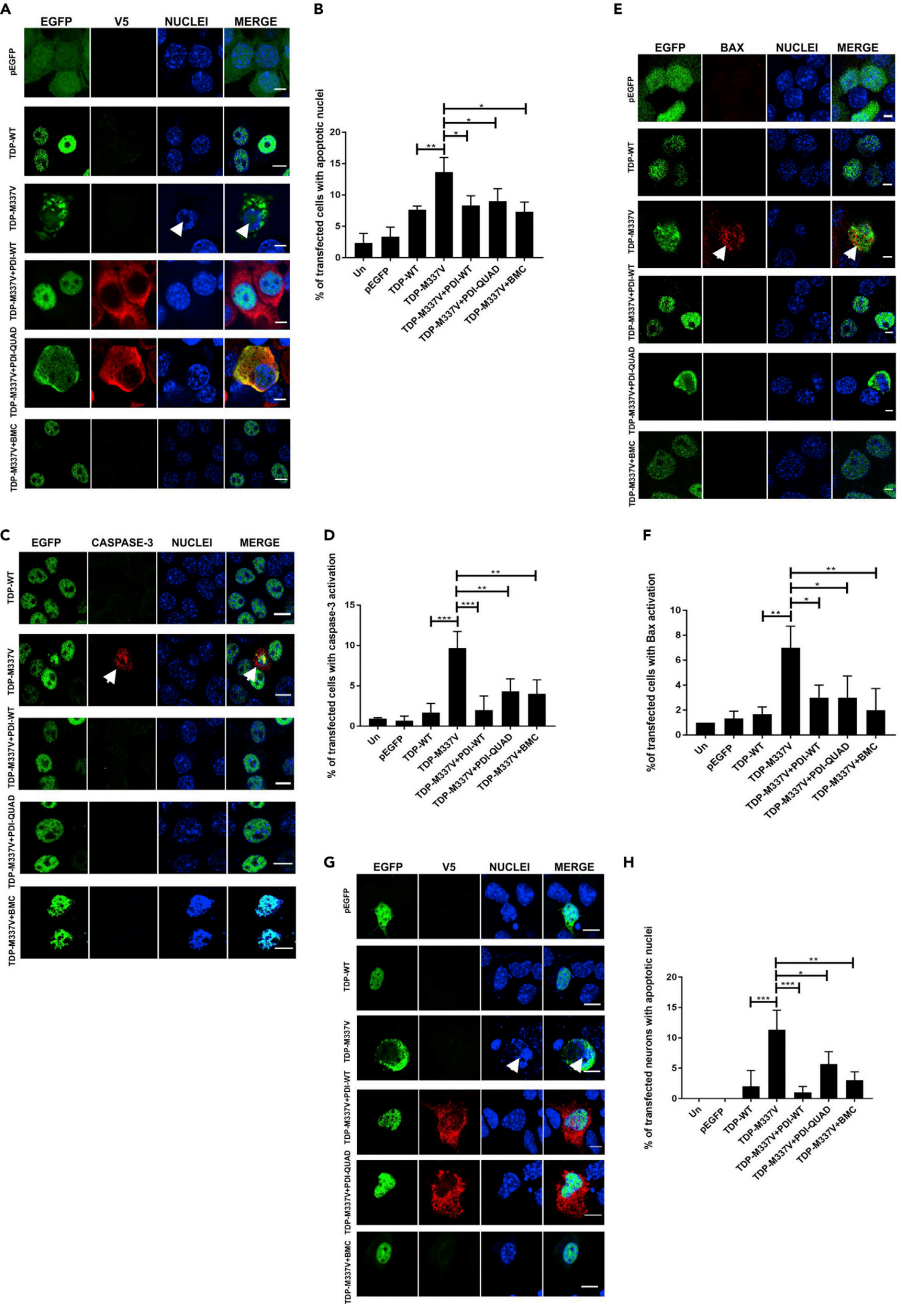
The Oxidoreductase and Chaperone Activities of PDI Are Protective against Mutant TDP-43 Induced Cell Death (A) Cells expressing EGFP (row 1), TDP-WT (row 2) or TDP-M337V (row 3), co-expressing PDI-WT or PDI-QUAD or BMC treated (rows 4, 5, 6), arrows represent apoptotic nuclei. (B) Over-expression of PDI-WT, PDI-QUAD, or BMC treatment with TDP-M337V resulted in significantly fewer cells with apoptotic nuclei compared with cells transfected with empty vector (∗p < 0.05). A significant difference was observed between TDP-WT and TDP-M337V expressing cells (∗∗p<0.01). (C) Immunocytochemistry using activated caspase-3 antibodies (red). Cells expressing TDP-WT (row 1) or TDP-M337V (row 2, arrows representing caspase-3 activation), co-expressing PDI-WT or PDI-QUAD, or treated with BMC (rows 3, 4, 5). (D) Over-expression of PDI-WT (∗∗∗p < 0.001), PDI-QUAD or treatment with BMC (∗∗p < 0.01), significantly decreased the proportion of cells with activated caspase-3. (E) Immunocytochemistry using anti-activated Bax antibodies (red). Cells expressing pEGFP (row 1), TDP-WT (row 2), or TDP-M337V (row 3, arrows representing Bax activation), co-expressing PDI-WT or PDI-QUAD, or treated with BMC (rows 4, 5, 6). (F) Over-expression of either PDI-WT or PDI-QUAD (∗p < 0.05), or treatment with BMC (∗∗p < 0.01), significantly decreased the proportion of cells with activated Bax compared with cells expressing empty vector. (G) Primary neurons expressing EGFP (row 1), TDP-WT (row 2) or TDP-M337V (row 3), co-expressing PDI-WT, PDI-QUAD, or treated with BMC (rows 4, 5, 6), arrows represent apoptotic nuclei. (H) Mutant TDP-M337V expression induced apoptosis (∗∗∗p < 0.001); however, over-expression of PDI-WT (∗∗∗p < 0.001), PDI-QUAD (∗p < 0.05) or administration of BMC (∗∗p < 0.01) resulted in significantly fewer neurons undergoing apoptosis. Scale bars: 8 μm in (A), (G), 10 μm in (C), 4 μm in (E).

We next examined whether the oxidoreductase or chaperone activity of PDI was protective against apoptosis induced by mutant TDP-43^M337V^ or mutant SOD1^A4V^, which was first quantified by the presence of apoptotic nuclei (condensed and fragmented DNA) using Hoechst staining (Walker et al., 2010, Cummings and Schnellmann, 2004) and by immunocytochemistry for the activated, cleaved form of caspase-3 (Parakh et al., 2018). Fragmented nuclei were rare in untransfected and EGFP cells (2%–3%) and were present in only 8% of cells expressing TDP-WT (Figure 7A). Significantly more cells expressing mutant TDP-43^M337V^ were undergoing apoptosis (14%, p < 0.01), but this proportion was markedly reduced by co-expression of PDI-WT or administration of BMC (8% and 7%, p < 0.05). Interestingly, PDI-QUAD also significantly reduced the proportion of cells with apoptotic nuclei (9%, p < 0.05, Figure 7B). Similarly, immunocytochemistry to detect activation of caspase-3 (Figure 7C) revealed that very few untransfected cells or those expressing EGFP or TDP-43-WT displayed caspase-3 activation (1%–2%). In contrast, 10% (p < 0.001) of TDP-43^M337V^-expressing cells displayed caspase-3 activation and were therefore apoptotic, but this was significantly reduced by co-expression of either PDI-WT (2%, p < 0.001) or PDI-QUAD, or by treatment with BMC (4%, p < 0.01, Figure 7D).These findings indicate that both the oxidoreductase and the chaperone activities of PDI are protective against apoptosis induced by mutant TDP-43.

A third marker of apoptosis was examined to confirm these findings. Upregulation of CHOP activates Bax, which results in its recruitment to mitochondria (Wolter et al., 1997). Hence, activation of Bax, detected using CHAPS buffer to lyse the nuclear membrane (Soo et al., 2009), was studied using immunocytochemistry and Hoechst staining of nuclei (Figure 7E). Negligible Bax activation was observed in control cells (untransfected, pEGFP or TDP-WT expressing, 1%–2%). More cells expressing mutant TDP-43^M337V^ displayed activation of Bax, indicating apoptosis (7%, p < 0.01), but this was significantly decreased by PDI-WT or PDI-QUAD co-expression (3%, p < 0.05) or treatment with BMC (2%, p < 0.01, Figure 7F).

Similar studies were carried out in primary mouse cortical neurons co-expressing TDP-EGFP and PDI-V5 as above, to validate these findings in more physiological conditions (Figure 7G). Few primary neurons expressing TDP-WT (2%) possessed apoptotic nuclei, unlike cells co-expressing mutant TDP-43^M337V^ with empty vector (11%, p < 0.001, Figure 7H). Co-expression of PDI-WT (1%, p < 0.001), or PDI-QUAD (6%, p < 0.05), and BMC treatment (3%, p < 0.01), significantly reduced the proportion of apoptotic nuclei compared with cells expressing mutant SOD1 only. Hence, these results confirm the findings obtained in cell lines and imply that both the oxidoreductase and chaperone activities of PDI contribute to its anti-apoptotic effect *in vitro* against TDP-43^M337V^.

#### Both the Oxidoreductase Activity and Chaperone Activity of PDI Are Protective against Apoptotic Cell Death Induced by Mutant SOD1

**Figure 8:**
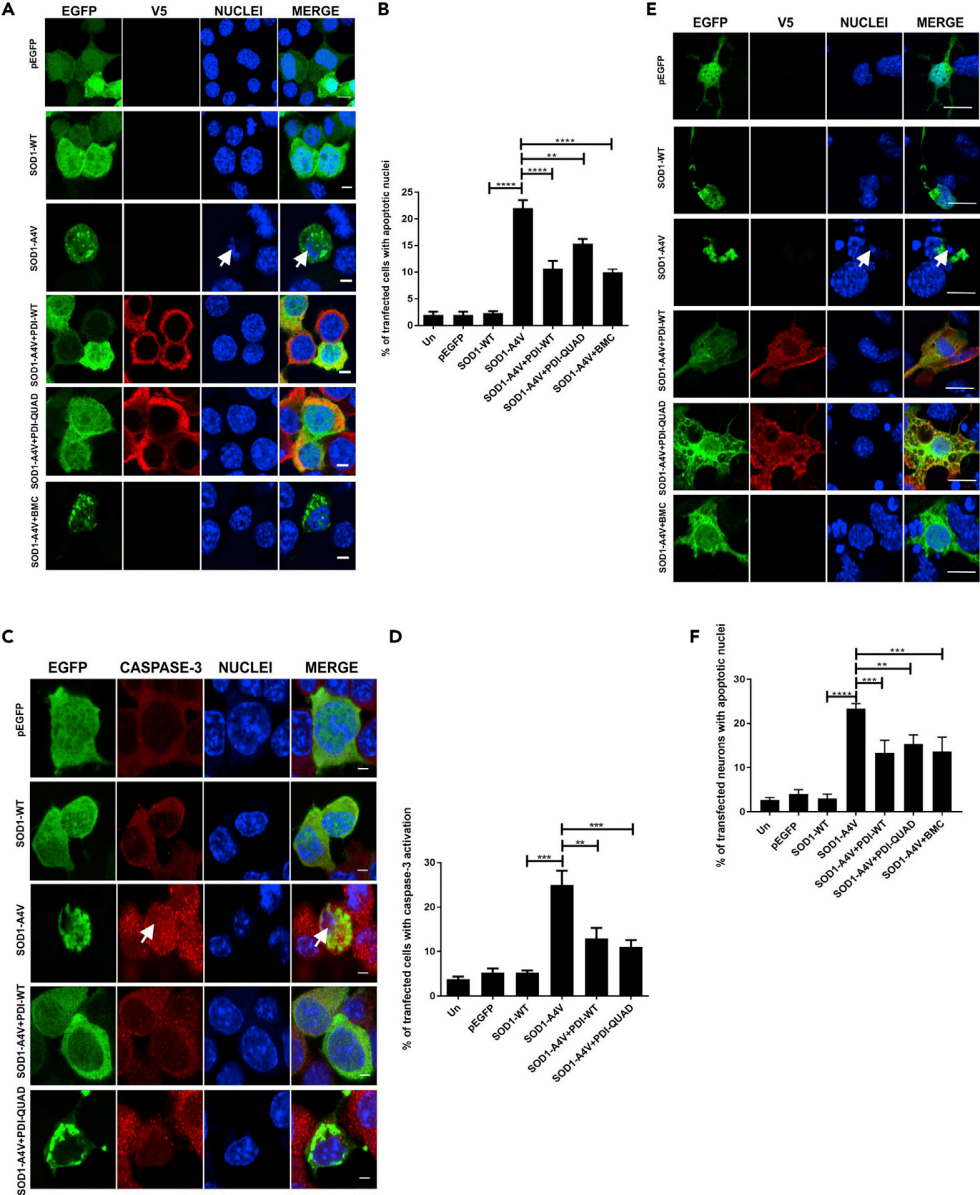
The Oxidoreductase and Chaperone Activities of PDI Are Protective against Mutant SOD1 Induced Cell Death (A) Neuro-2a cells expressing EGFP (row 1), SOD1-WT (row 2) or SOD1-A4V (row 3, condensed nuclei represented by white arrows), SOD1-A4V co-expressing PDI-WT or PDI-QUAD, or BMC treatment (rows 4, 5, 6). (B) Over-expression of PDI-WT (∗∗∗∗p < 0.0001), PDI-QUAD (∗∗p < 0.01) or BMC (∗∗∗∗p < 0.0001) with SOD1-A4V resulted in significantly fewer condensed apoptotic cells. A significant difference was observed between SOD1-WT and SOD1-A4V expressing cells (∗∗∗∗p<0.0001). (C) Immunocytochemistry using activated caspase-3 antibodies (red), white arrow represents caspase-3 activation. Cells expressing EGFP (row 1), SOD1-WT (row 2), SOD1-A4V (row 3), or co-expressing SOD1-A4V and PDI-WT or PDI-QUAD (rows 4, 5). (D) Over-expression of both PDI-WT (∗∗p < 0.01) and PDI-QUAD (∗∗∗p < 0.001) with SOD1-A4V significantly decreased the proportion of cells with activated caspase-3. (E) Primary neurons expressing pEGFP (row 1), SOD1-WT (row 2), or SOD1-A4V (row 3), co-expressing PDI-WT or PDI-QUAD, or treatment with BMC (rows 4, 5, 6). (F) Co-expression of PDI-WT (∗∗∗p < 0.001) or PDI-QUAD (∗∗p < 0.01) or treatment with BMC (∗∗∗p < 0.001) in SOD1-A4V expressing cells resulted in significantly fewer cells with apoptotic nuclei, identified by the presence of activated caspase-3. A significant difference was observed between SOD1-WT and SOD1-A4V (∗∗∗∗p<0.0001). Scale bars: 4 μm in (A) and (C), 10 μm in (E).

Similarly, we next examined whether the oxidoreductase or chaperone function is responsible for PDI's protective activity against mutant SOD1, using nuclear morphology and caspase-3 activation. Fragmented, apoptotic nuclei were rare in untransfected cells or those expressing EGFP only or SOD1-WT (2%), but significantly more were present in cells expressing mutant SOD1^A4V^ (22%, p < 0.0001) (Figure 8A). Co-expression of PDI-WT (11%) or treatment with BMC (10%, p < 0.0001) significantly reduced this proportion as previous (Walker et al., 2010), but interestingly, co-expression of PDI-QUAD also resulted in significantly fewer cells with apoptotic nuclei (15%, p < 0.01, Figure 8B). These data suggest that both the oxidoreductase and chaperone activities of PDI are protective against mutant SOD1^A4V^-induced apoptosis *in vitro*. Similar results were obtained following immunocytochemistry for activated caspase-3 (Figure 8C). Few control cells (untransfected, pEGFP or SOD1-WT-expressing cells) (≤5%) displayed immunoreactivity for caspase-3, but this proportion was significantly higher (25%, p < 0.001) in SOD1^A4V^-expressing cells, demonstrating that apoptosis was underway. Co-expression of PDI-WT (13%, p < 0.01) or PDI-QUAD (11%, p < 0.001, Figure 8D), as well as BMC treatment (12% (Figure S6, p < 0.05), also reduced the proportion of SOD1^A4V^ cells with caspase-3 activation.

To confirm these observations, primary mouse cortical neurons were co-transfected with SOD1-EGFP and PDI as above (Figure 8B). Cells with fragmented, apoptotic nuclei represented a minor population of untransfected neurons, or those expressing EGFP or SOD1-WT (≤5%), whereas consistent with previous studies (Parakh et al., 2018), almost 5-fold more mutant SOD1^A4V^-expressing cells were apoptotic (23%, p < 0.0001, Figure 8E). However, this proportion was significantly reduced by co-expression of either PDI-WT (13%, p < 0.001) or PDI-QUAD (15%, p < 0.01), or treatment with BMC (14%, p < 0.001, Figure 8F). Hence, together these results confirm that both the oxidoreductase and chaperone activities of PDI are protective against mutant TDP-43^M337V^ and mutant SOD1^A4V^-induced apoptosis *in vitro*.

### Depletion of Intracellular GSH Abrogates the Protective Activity of PDI against Pathological Forms of TDP-43 and SOD1 in Neuronal Cells

**Figure 9:**
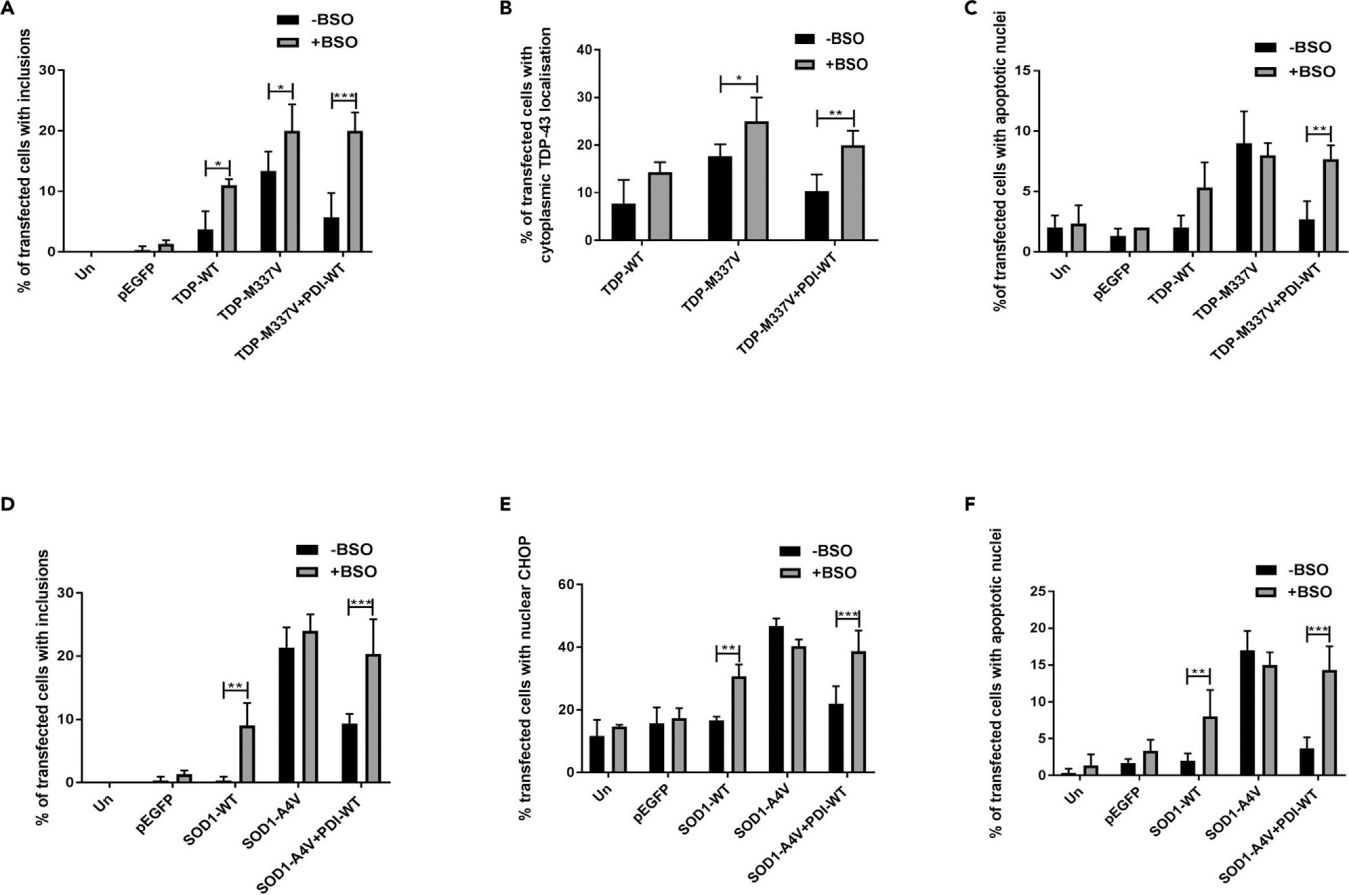
Depletion of Intracellular GSH Abrogates PDI's Activity against Mutant TDP-43 and Mutant SOD1 (A) BSO treatment resulted in more cells forming TDP-43 inclusions in cells expressing TDP-43 WT or mutant TDP-M337V (∗p < 0.05), and those co-expressing mutant TDP-M337V with PDI-WT (∗∗∗p < 0.001), compared with DMSO treatment. (B) BSO treatment resulted in more cells with cytoplasmic TDP-43 in populations expressing TDP-M337V (∗p < 0.05) and those co-expressing PDI-WT with TDP-M337V (∗∗p < 0.01), compared with DMSO treatment. (C) BSO treatment resulted in more cells with apoptotic nuclei in populations co-expressing PDI-WT (∗∗p < 0.01) with TDP-M337V compared with DMSO treatment. (D) BSO treatment resulted in significantly more cells forming inclusions when PDI-WT was co-expressed with SOD1-A4V (∗∗∗p < 0.001), and in SOD1-WT-expressing cells (∗∗p < 0.01), compared with DMSO treatment. (E) BSO treatment resulted in more cells expressing nuclear CHOP when PDI-WT was co-expressed with SOD1-A4V (∗∗∗p < 0.001) compared with empty vector, and in cells expressing SOD1-WT (∗∗p < 0.01) compared with DMSO treatment. (F) BSO treatment resulted in more cells with apoptotic nuclei in populations expressing SOD-WT (∗∗p < 0.01) and those co-expressing PDI-WT with SOD1-A4V (∗∗∗p < 0.001), compared with DMSO treatment.

To confirm these findings, we next modulated the cellular redox environment using BSO and then examined how this affected the protective activity of PDI. First, Neuro-2a cells expressing TDP-43-EGFP and PDI-WT (or empty vector) were treated with 75 μm BSO or DMSO at 24 h post transfection, and inclusion formation, TDP-43 mislocalization, and apoptosis were examined (Figures S7A–S7C). BSO treatment resulted in significantly more inclusions in cells expressing TDP-WT (4% versus 11%, p < 0.05) or mutant TDP-43^M337V^ (13% versus 20%, p < 0.05), or mutant TDP-43^M337V^ cells co-expressing PDI-WT (6%, 20% p < 0.001, Figure 9A), compared with DMSO-treated cells. In contrast, BSO did not alter the proportion of cells with cytoplasmic mislocalization in populations expressing TDP-WT (8% versus14%), whereas this percentage was increased in cells expressing mutant TDP-43^M337V^ (17% versus 25%, p < 0.05), and TDP-43^M337V^ with PDI-WT (10% versus 20%, p < 0.01, Figure 9B). Lastly, treatment with BSO did not alter the proportion of TDP-WT (5%) or mutant TDP-43^M337V^ (8%) cells undergoing apoptosis, but more apoptotic nuclei were present following BSO treatment in cells co-expressing TDP-43^M337V^ with PDI-WT (3%), compared with DMSO-treated cells (8%, p < 0.01, Figure 9C). Hence, these findings reveal that altering glutathione homeostasis using BSO ablates the protective activity of PDI against mutant TDP-43.

We then examined whether modulating the cellular redox environment with BSO affects PDI's protective activity against mutant SOD1^A4V^. Neuro-2a cells co-expressing SOD1-EGFP, and either empty vector or PDI-WT as above, were treated with BSO or DMSO 24 h post transfection. At 72 h post transfection, cells were examined for the presence of inclusions, ER stress, and apoptosis using microscopy (Figures S7D–S7F). BSO treatment did not significantly alter the proportion of inclusions formed in control cells (untransfected and EGFP only, <1%) or mutant SOD1^A4V^-expressing cells (21%–24%). However, interestingly, the percentage of inclusions formed in SOD1-WT cells was significantly increased following treatment with BSO (p < 0.01). Similar findings were obtained in cells co-expressing mutant SOD1^A4V^ with PDI-WT, where BSO treatment increased the proportion of cells bearing inclusions (9%, p < 0.01 and 20%, p < 0.001, Figure 9D). Hence, these data imply that BSO treatment induces the aggregation of SOD1-WT mutant SOD1^A4V^, and inhibits the protective activity of PDI.

ER stress was examined in mutant SOD1^A4V^-expressing cells by nuclear CHOP immunoreactivity. BSO treatment did not alter the proportion of control cells (untransfected or EGFP, 12%–17%) or cells expressing SOD1^A4V^ with nuclear immunoreactivity to CHOP (40%–47%). However, ER stress was significantly increased in BSO-treated cells co-expressing mutant SOD1^A4V^ with PDI-WT (39%, p < 0.001). Interestingly, significantly more nuclear CHOP immunoreactivity was present in SOD1-WT cells in the presence of BSO (31%) compared with DMSO-treated cells (p < 0.01 Figure 9E). Hence, BSO treatment ablates the protective activity of PDI against ER stress. Lastly, BSO treatment had no effect on the proportion of fragmented, apoptotic nuclei in control cells (untransfected, EGFP or SOD1-WT; 1%–3%) or those co-expressing mutant SOD1^A4V^ (15%–17%). However, more cells with apoptotic nuclei were detected in BSO-treated SOD1-WT cells or mutant SOD1^A4V^ cells co-expressing PDI-WT, compared with DMSO treatment (8%, p < 0.01 and 14%, p < 0.001, respectively, Figure 9F). This finding demonstrates that BSO inhibits PDI's protective activity against apoptosis and it induces toxicity in cells expressing SOD1-WT. Hence, these results together reveal that altering the GSH/GSSG pool impairs the protective activity of PDI against pathological forms of TDP-43 and SOD1. Interestingly, this also induced aggregation in TDP-WT cells, and aggregation, ER stress, and toxicity in cells expressing SOD1-WT.

### ALS-Linked Mutants (D292N and R300H) Lack the Protective Activity of PDI against Pathological Forms of TDP-43 and SOD1

**Figure 10:**
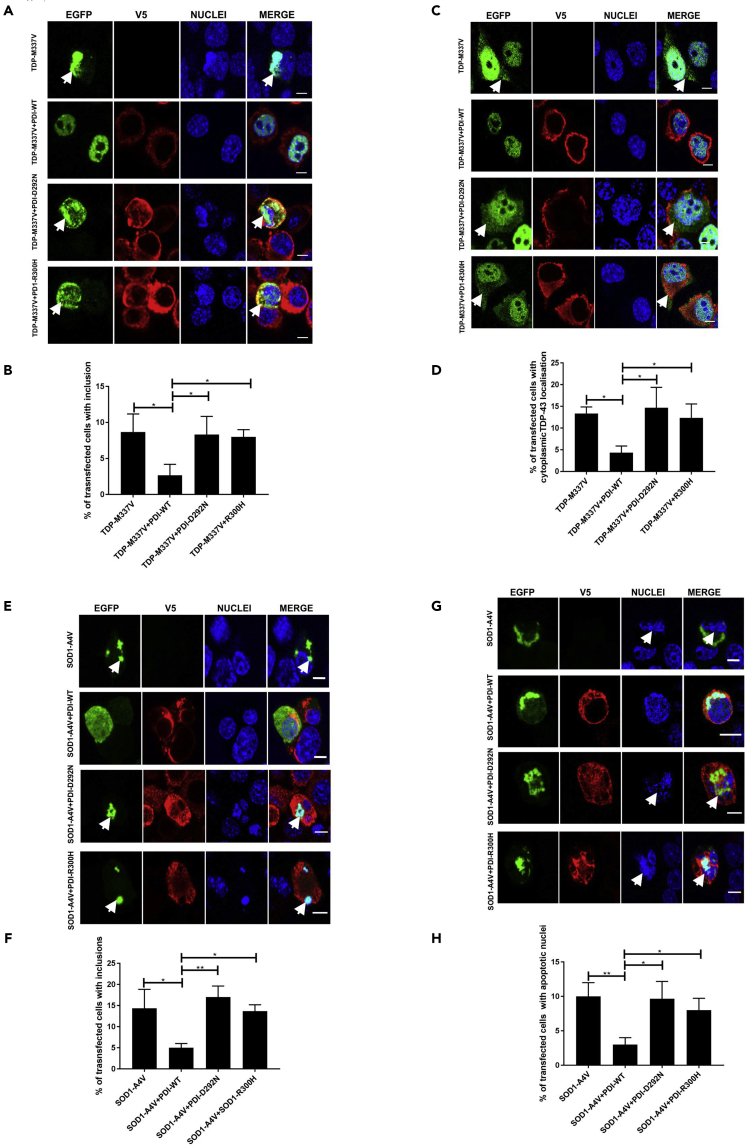
ALS-Linked PDI Mutants (D292N and R300H) Are Not Protective against Mutant TDP-43 and Mutant SOD1 (A) Cells expressing TDP-M337V with empty vector alone (row 1) or co-expressing PDI-WT, PDI-D292N or PDI-R300H (rows 2, 3, 4). (B) Significantly fewer cells formed inclusions when PDI-WT was co-expressed with TDP-M337V (∗p < 0.05) compared with PDI-D292N and PDI-R300H (∗p < 0.05). (C) Cells expressing EGFP-tagged TDP-M337V (row 1), or co-expressing PDI-WT, PDI-D292N or PDI-R300H (rows 2, 3, 4). (D) Co-expression of PDI-WT (∗p < 0.05) with mutant TDP-M337V significantly reduced the proportion of cells with cytoplasmic TDP-43 expression compared with those co-expressing PDI-D292N or PDI-R300H (∗p < 0.05). (E) Cells expressing SOD1-A4V (row 1) or co-expressing PDI-WT, PDI-D292N or PDI-R300H (rows 2, 3, 4). (F) Significantly fewer cells with inclusions were observed when PDI-WT was co-expressed with SOD1-A4V (∗p < 0.05) compared with either PDI D292N (∗∗p < 0.01) or PDI R300H (∗p < 0.01). (G) Neuro-2a cells expressing SOD1-A4V (row 1) or co-expressing PDI-WT, PDI-D292N or PDI-R300H (rows 2, 3, 4). (H) Over-expression of PDI-WT (∗∗p < 0.01) with SOD1-A4V resulted in significantly fewer cells with apoptotic nuclei compared with those expressing empty vector and those co-expressing PDI-D292N or PDI-R300H (∗p < 0.05). Scale bars: 6 μm in (A), (C), (E), and (G).

We next examined whether the ALS-associated mutants (D292N or R300H) affect the protective activity of PDI against mutant TDP-43 and mutant SOD1 (Woehlbier et al., 2016). We first examined mutant TDP-43 inclusion formation and cytoplasmic mislocalization. Co-expression of PDI-WT significantly reduced the proportion of mutant TDP-43^M337V^ cells forming inclusions (9%–3%, p < 0.05, Figure 10A), whereas co-expression of PDI-variants D292N or PDI-R300H (8%, Figure 10B) did not alter this proportion. Similarly, co-expression of PDI-WT resulted in significantly fewer cells with cytoplasmic TDP-43^M337V^ (13%–4%, p < 0.05, Figure 10C), whereas the ALS-mutants PDI-D292N (15%) and PDI-R300H (12%) had no effect, implying they were not protective against TDP-43 cytoplasmic mislocalization (Figure 10D). Hence, these data reveal that the ALS-linked PDI variants are not protective in cells expressing mutant TDP-43 inclusion formation and mislocalization to the cytoplasm.

Similarly, Neuro-2a cells were co-transfected with SOD1-EGFP and either PDI-WT or PDI-variants D292N or R300H, and inclusion formation and apoptosis were examined. Co-expression of PDI-WT with mutant SOD1^A4V^ significantly reduced the proportion of cells displaying inclusions (14%–5%, p < 0.05, Figure 10E), whereas co-expression of either PDI-D292N (17%) or PDI-R300H (14%) had no effect (Figure 10F). Hence, these data reveal that the ALS-linked PDI mutants do not protect against mutant SOD1 inclusion formation. Similarly, PDI-WT significantly reduced the proportion of mutant SOD1^A4V^ cells with apoptotic nuclei (10%–3%, p < 0.01, Figure 10G), whereas co-expression of PDI-D292N (10%) and PDI-R300H (8%) did not alter this proportion (Figure 10H). Hence, together these results demonstrate that the ALS-associated mutants lack the protective activity of PDI against pathological forms of TDP-43 and SOD1, thus providing new insights into their role in pathophysiology.

### The Oxidoreductase Activity of PDI and BMC Improves Motor Function in Zebrafish Expressing Mutant SOD1

**Figure 11:**
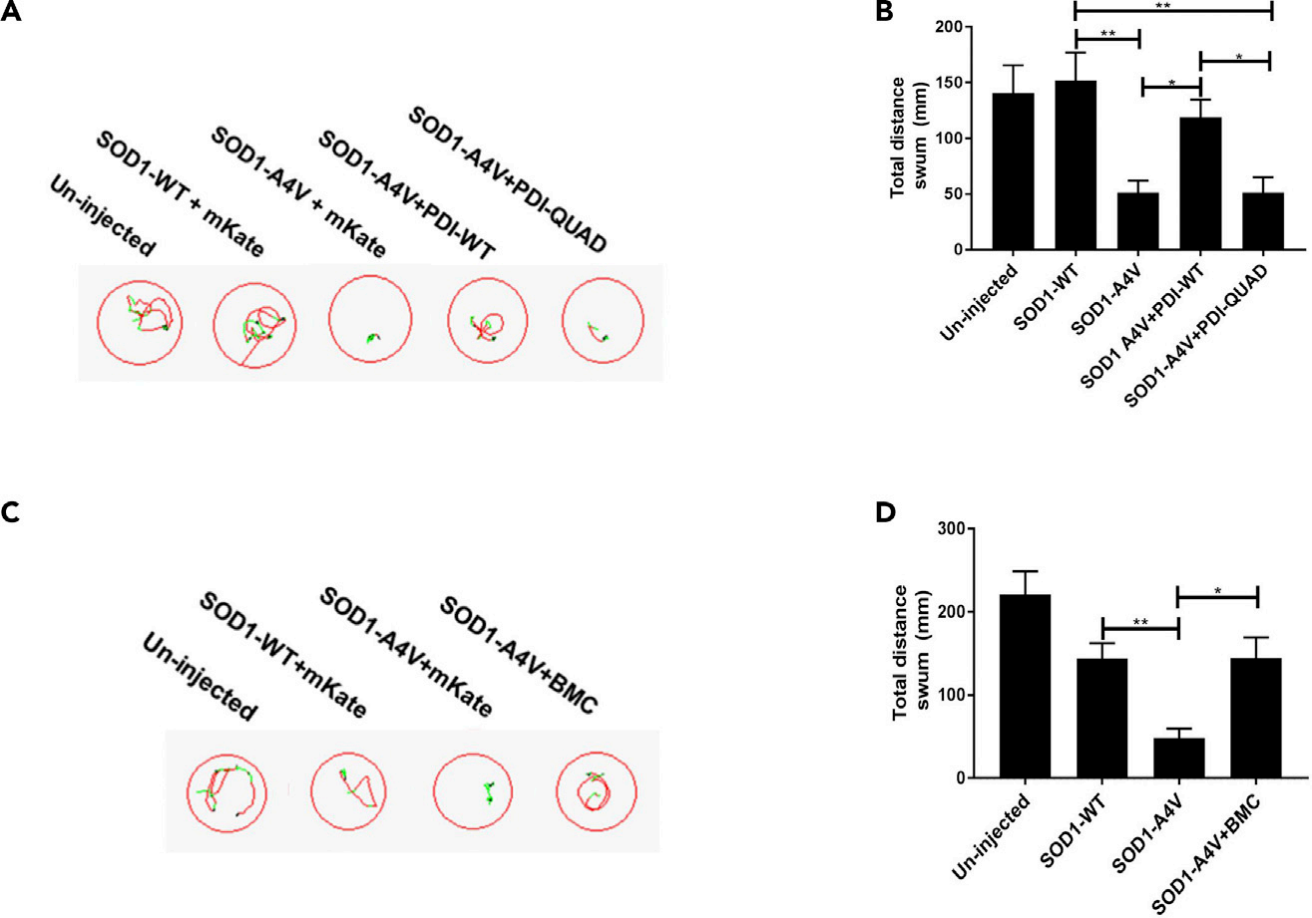
PDI's Oxidoreductase Activity Is Protective against Mutant SOD1-Induced Impaired Movement in Zebrafish Larvae (A) Representative images displaying the trajectory of movement of individual 48-hpf zebrafish larvae. Red lines indicate fast movement (>8 mm/s), green lines slow movement (3–8 mm/s), and black lines inactivity (<3 mm/s). (B) Total distance swum by zebrafish larvae in response to three flashes of light. Larvae injected with mutant SOD1-A4V traveled a significantly shorter distance in response to the flashes of light than un-injected larvae or those expressing SOD1-WT (∗∗p = 0.0031). Zebrafish larvae co-expressing SOD1^A4V^ and PDI-WT swam significantly longer (∗p < 0.021) distances than those expressing mutant SOD1-A4V, or co-expressing SOD1-A4V and PDI-QUAD, n > 31 per group. (C) Representative images displaying the trajectory of movement of individual larvae in response to three flashes of light. Treatment with BMC increased the distance traveled by larvae expressing mutant SOD1-A4V. (D) Un-injected zebrafish larvae and larvae expressing SOD1-WT swam significantly longer distances than those expressing SOD1-A4V (∗∗p < 0.0040). Treatment with BMC resulted in a significant increase in the distance swum by the SOD1^A4V^ larvae (∗p < 0.021) compared with treatment with DMSO alone, n > 28 per group.

To assess the protective effects of PDI in an *in vivo* setting, we next examined zebrafish that transiently express human SOD1^A4V^. It has previously been demonstrated that these zebrafish develop shortened motor axons (Lemmens et al., 2007) that correlate with impaired movement (Robinson et al., 2019). Human SOD1 mRNA (WT or A4V) was co-injected with mRNA for the fluorophore mKate2, PDI-WT-mKate2, or PDI-QUAD-mKate2. At 48 h post fertilization (hpf) behavioral testing was performed to examine motor impairment (Figure 11A). The distance swum by zebrafish co-expressing SOD1^A4V^ with mKate2 in response to a flash of light was significantly shorter than un-injected controls (p = 0.0279) and those co-expressing SOD1-WT with mKate2 (p = 0.0031, Figure 11B). In comparison, zebrafish expressing SOD1^A4V^ and PDI-WT-mKate2 swam significantly longer distances than those co-expressing SOD1^A4V^^with^ Kate2 (p = 0.0151) and similar distances to those co-expressing SOD1-WT with mKate2 (p > 0.9999). Moreover, those co-expressing SOD1^A4V^ with PDI-QUAD-mKate2 swam similar distances to those co-expressing SOD1^A4V^ and mKate2 (p > 0.9999), and significantly shorter distances than both those expressing SOD1^A4V^ and PDI-WT-mKate2 (p = 0.0110) and SOD1-WT (p = 0.0022), indicating that unlike PDI-WT, PDI-QUAD did not prevent impaired movement of the zebrafish.

To confirm these findings, SOD1^A4V^ and mKate2-injected embryos were also treated with BMC (12.5 μM) or DMSO as a control (Figure 11C). The SOD1^A4V^ zebrafish swam significantly shorter distances than both SOD1-WT and mKate2 or un-injected controls (p < 0.0040) as expected, whereas treatment with BMC resulted in a significant increase in the distance swum by the SOD1^A4V^ and mKate2 embryos (p = 0.021, Figure 11D). Together, these results demonstrate that the oxidoreductase activity of PDI is protective against ALS-relevant phenotypes *in vivo*.

## Discussion

PDI is the archetype of a family of chaperones that perform two major functions: (1) the formation and isomerization of native disulfide bonds in proteins via oxidoreductase activity, and (2) general chaperone activity. As ALS is a protein misfolding disorder, we initially hypothesized that the chaperone activity of PDI would be protective against neurodegeneration. However, in contrast, here we demonstrate that the oxidoreductase activity of PDI mediates its protective activity against two major pathological proteins linked to ALS, SOD1 and TDP-43. Surprisingly, the chaperone activity was only protective against apoptosis and could not prevent the other ALS phenotypes examined, even protein misfolding. Importantly, TDP-43 is misfolded in almost all ALS cases, thus placing PDI onto the broad pathophysiology of ALS. For these studies we used the PDI-QUAD mutant, and we first confirmed that PDI-QUAD lacks the normal oxidoreductase activity of PDI, using a redox biosensor. This is consistent with previous studies showing that mutations in one or both of the catalytic sites of PDI retain its chaperone activity but lead to loss of its redox-dependent disulfide isomerization functions (Whiteley et al., 1997, Hashimoto et al., 2008). The redox activity of PDI-WT was found to be protective against protein unfolding, the formation of inclusions, mislocalization to the cytoplasm, ER stress, ER-Golgi transport defects, and apoptosis, induced by pathological forms of both TDP-43 and SOD1 in neuronal cell lines and primary neurons. Furthermore, the oxidoreductase activity of PDI was protective against motor impairment in zebrafish models of ALS. These findings were confirmed by pharmacological approaches, using both BMC, a small molecule mimic of the active site with a similar redox potential to PDI, and BSO, which modulates the redox state and thus inhibits the normal function of PDI. We also demonstrate that ALS-associated PDI variants (D292N and R300H) (Woehlbier et al., 2016), which were previously found not to directly induce toxicity themselves, lack the oxidoreductase property of PDI and were not protective against mutant SOD1 or mutant TDP-43. Hence, this finding provides unique insights into the role of these variants in ALS, further emphasizing the role of redox-modulated PDI in ALS and also adding clinical relevance to this study.

These results therefore imply that the oxidoreductase activity of PDI centrally regulates the neuronal redox environment, controlling multiple cellular phenotypes that dysfunction in ALS. They also suggest that the redox activity of PDI can overcome cellular defects induced by mutant ALS proteins. Hence, these findings implicate redox homeostasis centrally in ALS, with a much broader role in neurodegeneration than previously recognized. Redox homeostasis involves specific oxidation/reduction reactions that go beyond the commonly described term 'oxidative stress', because it regulates a myriad of processes that are linked to signaling and metabolism. The importance of redox homeostasis is not limited to regulating reactive oxygen species (ROS) levels but involves the transfer of electrons between redox proteins and the cycling of such redox-regulated proteins from their oxidized to reduced state (Franco and Vargas, 2018). Our findings raise the possibility that harnessing the oxidoreductase property of PDI could be used for designing therapeutic agents that could be trialed in ALS.

Modulating the intracellular pool of glutathione using BSO led to loss of the protective function of PDI, implying that when redox conditions are dysregulated in ALS, this safeguarding property of PDI is further compromised. In BSO-treated cells co-expressing PDI and mutant SOD1 or mutant TDP-43, increased inclusion formation, ER stress, TDP-43 mislocalization, and neuronal cell death were detected. These findings imply that redox dysregulation leads to alterations in the enzymatic activity of PDI, such as the previously described S-nitrosylation (Walker et al., 2010). Importantly, we also demonstrate that when treated with BSO, wild-type forms of both SOD1 and TDP-43 form inclusions and SOD1-WT induces ER stress and apoptosis, again highlighting redox dysregulation as a central process underlying neurodegeneration in ALS. Consistent with our findings, oxidized SOD-WT misfolds, forms aggregates, develops a similar conformation as the mutant, and gains toxic functions, including mitochondrial dysfunction and aggregation *in vitro* (Guareschi et al., 2012, Bosco et al., 2010). Similarly, aberrant disulfide cross-linking leads to misfolding and subcellular mislocalization of TDP-43 (Cohen et al., 2012, Barmada et al., 2010).

Proteostasis refers to the mechanisms regulating protein biogenesis, folding, trafficking, and degradation, that maintain cellular homeostasis (Klaips et al., 2018). This study provides evidence that PDI has a broad protective role against proteostasis mechanisms associated with ALS (protein folding, trafficking, and ER homeostasis), and they emphasize the relevance of the redox-dependent function of PDI in this process. PDI normally catalyzes the efficient folding of newly synthesized proteins; hence, it plays an important role in protein quality control. However, it should be noted that the protective functions of PDI can be modulated by its subcellular location, levels of ER stress, cellular redox environment, and post-translational modifications. In rat models of Huntington disease, the presence of PDI at the ER-mitochondrial associated membranes has even been shown to induce apoptosis (Hoffstrom et al., 2010). Post-translational modifications of PDI such as S-nitrosylation (SNO-PDI) also accentuate the misfolding of synphilin in Parkinson's disease (Forrester et al., 2006), and SNO-PDI increases mutant SOD1 misfolding via incorrect disulfide cross-linking, leading to neuronal apoptosis (Jeon et al., 2014). This implies that PDI may have a dual-edged role in neurodegenerative diseases, and in some instances, it may even be harmful (Parakh and Atkin, 2015).

In this study, the redox activity of PDI prevented the deleterious cellular features of pathogenic forms of SOD1 and TDP-43 and restored motor function in a zebrafish model of ALS, a validated model for dissecting mechanisms of neurodegeneration in ALS (Kabashi et al., 2010, Patten et al., 2014). Our findings also therefore place redox dysregulation centrally as a pathogenic process in ALS.

Disulfide bonds dictate protein structure, and protein misfolding is an intrinsic propensity of most proteins (Herczenik and Gebbink, 2008). Misfolded proteins normally display buried hydrophobic regions at their surfaces, and they can accumulate into protein aggregates that are closely associated with toxicity (Sweeney et al., 2017). Therefore, enzymes that can refold aberrantly folded proteins and prevent the formation of protein aggregates may be important therapeutically. Importantly, PDI was shown to have a key role in oxidative protein folding, since its deletion in mammalian cells results in delayed disulfide bond formation (Rutkevich et al., 2010). Mutant SOD1 and mutant TDP-43 both form aberrant, non-native disulfide bonds, which have been detected in ALS patient tissues; they may also contribute to aggregation under cellular stress conditions *in vitro* (Bosco et al., 2010, Cohen et al., 2012). Recent studies have demonstrated that small oligomeric forms of SOD1 are toxic rather than the large aggregates themselves (Proctor et al., 2016, Zhu et al., 2018). Therefore, in this study, we used a novel fluorogenic dye to specifically quantitate the load of unfolded proteins as well as examine the formation of SOD1 aggregates (Chen et al., 2017). This is a unique tool to explore the relationship between protein misfolding and the cellular backlog of unfolded proteins. Our data imply that the active site cysteine residues in PDI, as well as BMC treatment, prevent proteins from unfolding and thus inhibit inclusion formation and misfolding. Treatment with BMC induced a significant intracellular oxidation in cells expressing the redox biosensor alone or those co-expressing PDI-WT and PDI-R300H. In contrast, cells expressing PDI-D292N or PDI-QUAD proved refractory to the effect of BMC. This intriguing behavior may be tentatively ascribed to some inhibitory effect of these mutants on BMC activity. This possibility should be investigated further because patients with ALS presenting with this mutation may be refractory to pharmacological intervention with molecules enhancing protein disulfide bond formation. However, an alternative explanation for these findings is that BMC is neuroprotective in a manner dependent on the activity of PDI. Similarly, in yeast models, PDI mutants containing deletions in the active site motif delayed disulfide bond formation (LaMantia and Lennarz, 1993).

We also found that the chaperone activity of PDI was not protective against ER dysfunction, ER stress or inhibition of ER-Golgi transport, in cells expressing mutant SOD1 or mutant TDP-43. ER stress is observed in the early stages of disease in transgenic SOD1^G93A^ mice, in those motor neurons that degenerate first, highlighting a central role in neurodegeneration (Saxena et al., 2009). Furthermore, inhibition of ER-Golgi transport is a probable upstream trigger of ER stress in cells expressing mutant SOD1 or mutant TDP-43 (Atkin et al., 2014, Soo et al., 2015). The three PDI mutants lacking redox activity (PDI-QUAD, D292N, and R300H) were found not to be protective against ER stress induced by mutant SOD1 or mutantTDP-43. Hence, this suggests that the oxidoreductase activity of PDI, which mediates the isomerization and formation of disulfide bonds in other substrate proteins, inhibits the levels of stress within the ER. Although the substrate-binding b and b’ domains of PDI are essential for its chaperone activity by their binding to hydrophobic regions of misfolded proteins, complex reactions that involve extensive conformational changes in the substrate require all of the PDI domains together (Klappa et al., 1998). Similarly, both D292N and R300H mutations are present in the substrate-binding b′ domain, and substrate binding is known to be a redox-dependent process (Woehlbier et al., 2016). It was previously suggested that these mutations affect the binding-release cycle of substrates (Woehlbier et al., 2016). Hence, this may explain why the chaperone activity of PDI alone cannot protect against the load of misfolded proteins associated with ALS, and consequent induction of ER stress. Since both mutant SOD1 (Jeon et al., 2014) and mutant TDP-43 (Walker et al., 2013, Parakh et al., 2018) are thought to interact with PDI, this could sequester PDI and perturb its chaperone activity, rendering it non-functional. This possibility should therefore be investigated in the future.

Several mechanisms associated with ALS, including oxidative stress, heat shock, ER stress, as well as mutations in the NLS region, result in the re-distribution of TDP-43 from the nucleus to the cytoplasm (Nonaka et al., 2009). Interestingly, we also found that the oxidoreductase activity of PDI, but not its chaperone function, was protective against mutant TDP-43 mislocalization to the cytoplasm. This finding is consistent with evidence linking redox dysregulation to TDP-43 mislocalization (Cohen et al., 2012, Barmada et al., 2010, Winton et al., 2008). It also raises the question of where PDI is exerting its protective activity. Although PDI is primarily localized in the ER, PDI has also been described in the cytoplasm, in the nucleus, and at the cell surface (Turano et al., 2002). In ALS, mutant SOD1 and mutant TDP-43 are mainly cytoplasmic and not present within the ER lumen (Soo et al., 2015, Nishitoh et al., 2008), implying that PDI is protective in the cytoplasm. Consistent with this notion, a recent study identified a novel “protein reflux” system that relocates PDI from the ER to the cytosol during ER stress, distinct from ER-associated degradation (ERAD). Hence, this process may increase the cytoplasmic pool of PDI in ALS, from where its redox activity can prevent mislocalization of mutant TDP-43 to the cytoplasm (Igbaria et al., 2019). Thus, our data imply that PDI prevents misfolding of mutant TDP-43 and mutant SOD1 in the cytoplasm, rather than at the ER, and this may be the primary location where the thiol-disulfide is protective.

The previously described PDI variants, D292N and R300H (Woehlbier et al., 2016), were found here to lack the oxidoreductase activity of PDI-WT. Consistent with the PDI-QUAD results, they were not protective against mutant SOD1 and mutant TDP-43-induced inclusion formation, apoptosis, or mislocalization of mutant TDP-43 to the cytoplasm. These variants are over-represented in patients with ALS compared with control subjects, and it has been previously suggested that they may be risk factors or phenotypic modifiers of disease. Both D292N and R300H mutations are present in the substrate-binding b′ domain, and substrate binding is known to be a redox-dependent process (Woehlbier et al., 2016). Furthermore, R300H was found to possess decreased oxidative catalytic activity compared with PDI-WT (Woehlbier et al., 2016), consistent with our findings. Although some abnormalities have been previously observed when D292N and R300H were expressed in zebrafish, surprisingly, these mutations were not neurotoxic when expressed in cell culture and normal homeostasis in the ER was also maintained (Woehlbier et al., 2016). However, our findings now provide insights into these observations, because they imply that, despite the lack of direct toxicity, D292N and R300H prevent PDI from performing its normally protective functions against the proteins that typically misfold in ALS. Hence, it is tempting to speculate that this is why these variants act as risk or modifying factors in ALS. Single-nucleotide polymorphisms (SNPs) and SNP haplotypes in the *P4HB* gene, encoding PDI, have also been associated with both fALS and sALS (Kwok et al., 2013, Yang and Guo, 2016). However, these SNPs are intronic, so it remains unclear how they are linked to toxicity.

It is interesting to note, however, that the PDI-QUAD mutant retained the protective activity against apoptosis. Although the PDI active site mutants cannot perform the disulfide isomerization functions (Whiteley et al., 1997, Hashimoto et al., 2008), they retain its chaperone activity, and in yeast, they do not impact on cellular viability (LaMantia and Lennarz, 1993), consistent with our findings. It could also be speculated that this mechanism is used by the cell as a last resort to restore cellular homeostasis. Hence, this process may result in the chaperone activity of PDI being protective against apoptosis, as observed here. However, since both the PDI variants possess a mutation in the b’ domain, which is essential for substrate binding, it could be speculated that they lose their chaperone activity and hence did not protect against apoptosis.

PDI has distinctive properties that make it an effective catalyst, such as conformational flexibility, rapid ligand exchange, broad substrate specificity, and the ability to differentiate between unfolded and partially folded proteins (Irvine et al., 2014). The PDI family is unique among protein chaperones because members of this family reduce aggregation of misfolded proteins by directing them to ERAD for degradation, or by isomerizing disulfide bonds present within protein substrates until the correct confirmation is achieved. This dual property of PDI may be particularly important in ALS (and other neurodegenerative conditions) because disturbances to autophagy and proteasomal functions are associated with pathogenesis, which would further exacerbate protein misfolding (Ramesh and Pandey, 2017).

The findings of this study therefore imply that one of the normal cellular functions of PDI is to protect against redox-dependent pathological processes induced by proteins associated with neurodegeneration that are prone to misfold. However, owing to unfavorable cellular conditions, such as redox dysregulation leading to oxidative stress, PDI becomes non-functional. Cysteine residues are required for the normal oxidoreductase function of PDI, but they are also susceptible to aberrant modification by oxidative stress (Conway and Harris, 2015), leading to loss of PDI function. These data suggest that the oxidoreductase activity of PDI is pivotal for its protective function in ALS. Furthermore, dysregulation in the cellular redox environment can lead to adverse cellular defects. This raises the possibility that therapeutic elevation of the oxidoreductase activity of PDI, either by increasing the cellular levels of PDI or by administration of small molecular mimics of PDI activity, may have potential for the treatment of ALS and related neurodegenerative disorders associated with protein misfolding. Understanding the key features that mediate the protective activity of PDI may therefore facilitate future studies that aim to develop therapeutic strategies to combat protein misfolding disorders. Our study also implicates redox homeostasis as the key mechanism controlling the formation of phenotypes typical of ALS. Hence, regulation of the motor neuronal redox environment may contribute more significantly to ALS than previously realized.

### Limitations of the Study

The current studies incorporated the use of cell culture and zebrafish models overexpressing mutant proteins linked to ALS. The rationale for using these models was to gain a simplified, yet accelerated, view of the pathological consequences of expression of these mutant proteins to investigate the effect of PDI. However, it should be noted that transient transfections result in high levels of expression of mutant proteins, which may be non-physiological and therefore give rise to phenotypes not directly relevant to ALS. Investigating cellular pathologies using stable cell lines expressing PDI may eliminate aberrantly high levels of expression. Another caveat that should be mentioned is that transport of VSVG^ts045^ from the ER to Golgi was the only method used to investigate cellular trafficking dysfunction, and this uses an overexpressed, non-physiological marker. Therefore, other alternative approaches are warranted in the future, such as examining overall protein secretion in neurons or additional ER/Golgi transport assays. Similarly, the use of transgenic ALS rodent models is another approach that would allow us to further confirm our *in vivo* findings in zebrafish. These additional experiments would further strengthen our findings that the redox activity of PDI plays a central role in ALS.

### Resource Availability

#### Lead Contact

Further information and requests for resources and reagents should be directed to and will be fulfilled by the Lead Contact, Dr Sonam Parakh (sonam.parakh@mq.edu.au).

#### Materials Availability

Plasmid generated in this study have been deposited to Addgene [PDI-QUAD-V5-pcDNA3.1 Addgene-153550]. All methods can be found in the accompanying Transparent Methods supplemental file.

#### Data and Code Availability

The datasets used and/or analyzed during the current study are available from the lead author on reasonable request.

## Methods

All methods can be found in the accompanying Transparent Methods supplemental file.

## Supplemental Information

Document S1. Transparent Methods and Figures S1–S7
