# Supplementary material for: The Redox Activity of Protein Disulfide Isomerase Inhibits ALS Phenotypes in Cellular and Zebrafish Models
Source: iScience. 2020 Apr 25;23(5):101097. doi: 10.1016/j.isci.2020.101097 (PMC7240177; doi:10.1016/j.isci.2020.101097)
Supplement: Document S1. Transparent Methods and Figures S1–S7 [file mmc1.pdf]

iScience, Volume *23*

Supplemental Information

The Redox Activity of Protein Disulfide Isomerase Inhibits ALS Phenotypes in Cellular and Zebrafish Models

Sonam Parakh, Sina Shadfar, Emma R. Perri, Audrey M.G. Ragagnin, Claudia V. Piattoni, Mariela B. Fogolín, Kristy C. Yuan, Hamideh Shahheydari, Emily K. Don, Collen J. Thomas, Yuning Hong, Marcelo A. Comini, Angela S. Laird, Damian M. Spencer, and Julie D. Atkin

Supplemental Information

Transparent materials and methods

Cell lines

Mouse neuroblastoma Neuro-2a cell lines (ATCC cell line CCL-131) were maintained in Dulbecco's modified eagle medium (DMEM) with 10% fetal calf serum (FCS) and incubated at 37°C with 5% CO_2.

Cortical neuronal cultures

All animal husbandry and experimental procedures were performed in compliance with the Animal Ethics Committee, Macquarie University, NSW, Australia (ARA 2017/020-5) and the Internal Biosafety Committee, Macquarie University (NLRD 5974-52019597412350). Primary neurons were harvested from the cortex of C57BL/6 mouse embryos at embryonic day 16-18. The procedure for culture of primary neurons was as described previously (Parakh et al., 2018). Briefly, cortical tissue was dissected in Hanks' Balanced Salt solution (HBSS, Gibco) and digested in 10 units/ml papain (Sigma) in 2 mg/ml L-cysteine (Sigma) and 0.5 mM EDTA, pH 8 (Sigma) in DMEM, for 15 min at 37°C. Subsequently, cells were triturated and dissociated, then resuspended in medium (DMEM, 10% FBS, 100 µg/ml penicillin-streptavidin) and seeded for 1 hr on glass coverslips, previously coated with poly-D-lysine. Cells were then incubated in neuronal medium (Neurobasal medium supplemented with 2% B27 supplement (Gibco), 1% Glutamax (Gibco) and 100 µg/ml penicillin-streptavidin) at 37°C and 5% CO_2.

Expression constructs

Previously generated pcDNA3.1(+) constructs encoding wild-type PDI (PDI-WT) were a generous gift from Professor Bulleid (Jessop et al., 2009). PDI-QUAD was generated from PDI-WT pcDNA3.1 (+) using two native active sites (CGHC, CGHC) tagged with V5, and mutated (to SGHS, SGHS) using site directed mutagenesis (Addgene 153550). The SOD1-WT and SOD1-A4V constructs encoding EGFP-tagged human SOD1 at the C-terminus were as described previously (Turner et al., 2005). TDP-WT and mutant TDP-43 Q331K constructs encoding EGFP-tagged human TDP-43 at the N-terminus were provided by Professor Benjamin Wolozin, Boston University, USA. Wild-type TDP-43 and mutant TDP-43 M337V in pCMV6-AC-GFP to allow expression of human TDP-43 with a C-terminal GFP tag were as previously described (Farrawell et al., 2015). TDP-WT and mutant TDP-43 Q331K in pmCherry.N1 to allow expression of human TDP-43 with a C-terminal mCherry tag were as previously described (Walker

et al., 2013). PDI-D292N and PDI-R300H-V5 tagged constructs were kindly provided by Professor Claudio Hetz (Woehlbier et al., 2016). The mutant vesicular stomatitis viral glycoprotein VSVG^{ts045} fused to EGFP in pEGFP-C1 vector was a kind gift from Dr Jennifer Lippincott-Schwartz (National Institutes of Health, Bethesda, USA).

Cell culture and transfection

Transfections were performed using LipofectamineTM2000 (Invitrogen) according to the manufacturer's protocol. Cells were co-transfected with SOD1 or TDP-43 (EGFP) and PDI-V5 constructs and observed 72 hr post-transfection using either confocal or fluorescence microscopy. For primary neurons, after 5-7 days *in vitro*, cultured cortical neurons were co-transfected for 72 hr with 1 µg of pEGFP-N1 vector, EGFP-tagged wild-type or mutant SOD1^{A4V} or EGFP-tagged wild-type or mutant TDP-43^{M337V} with 1 µg of plasmid encoding V5-tagged PDI's or empty vector pcDNA3.1, using 2 µg Lipofectamine™ 2000 (Invitrogen), following the manufacturer's instructions.

Immunoblotting

Cell lysates were collected in cold TN buffer (50 mM Tris–HCl pH 7.5 and 150 mMNaCl, pH 7.6) with 1% Triton-X 100, protease inhibitors (pi, 1:1000), phosphatase inhibitors (ppi, 1:100) and Phenylmethanesulfonyl fluoride [PMSF, Sigma #P7626–250MG (1: 500)], then incubated on ice for 15 min and stored at −20°C overnight. Samples were sonicated for 10× 1″ bursts and centrifuged at 100,000*g* at 4°C for 30 min to obtain the SDS-soluble fraction. Protein concentrations of cell lysates were determined using the BCA protein assay (Thermo scientific) by comparison with BSA standards. Protein samples (20 µg) were electrophoresed through 12% SDS–polyacrylamide gels and transferred to nitrocellulose membranes. Membranes were blocked with 5% skim-milk in Tris-buffered saline (pH 8.0) for 30 min, then incubated with the appropriate primary antibodies at 4°C for 16 hr: anti-GFP (1:500, Abcam), anti-V5 (1:1000, Abcam), rabbit anti-TDP-43, (1:1000, Cosmo) or anti-β-actin (1:1000, Sigma). Membranes were incubated for 1 hr at room temperature with secondary antibodies (1:4000, HRP-conjugated goat anti-rabbit, or goat anti-mouse, Chemicon), and detected using ECL reagent (Bio-Rad). Precision Plus Protein™ Dual Color Standard molecular weight markers were used (Bio-Rad). Quantitation of blots was performed by densitometry using ImageJ (NIH). SOD1 expression blots were cut in to strips to save on reagents. The appropriate region was selected by observing the position of the molecular weight markers (Figure 4A).

Immunofluorescence and microscopy

Neuro-2a cells were fixed in 4% paraformaldehyde, permeabilized with 0.1% Triton-X in phosphate buffered saline (PBS), blocked with 3% BSA in PBS, followed by incubation with mouse anti-CHOP (1:50, Santa Cruz), rabbit anti-XBP-1 (1:20, Santa Cruz), mouse anti-V5 (1:250, Abcam) rabbit anti-active cleaved-caspase-3 (1:20, Cell Signalling), or rabbit anti-Bax (1:200, BD Biosciences) antibodies in PBS at 4°C overnight. The secondary antibodies, AlexaFluor 568-conjugated rabbit anti-mouse IgG (1:250) or goat-anti-rabbit IgG AlexaFluor 568, were added for 1 hr and incubated in the dark at room temperature. After washing with PBS, staining of nuclei was performed using Hoechst stain 33342 (Invitrogen, nuclei), FITC (GFP fluorescence) and TRITC (red fluorescence) filters were used for viewing cells and the images were taken using a Zeiss confocal Axioimager microscope or LSM 880 Zeiss confocal microscope. In dual-channel imaging, photomultiplier sensitivities and offsets were set to a level at which bleed-through effects from one channel to another were negligible. Immunocytochemistry for XBP-1 in TDP-43 expressing cells was performed at 18 hr post-transfection.

VSVG transport assay

Neuro-2a cells were transiently transfected with the appropriate plasmid and VSVG^{ts045}-mCherry. Cells were incubated at 40˚C under 5% CO2 for 12.5 hr for SOD1 and 16 hr for TDP-43. The cells were then treated with cDMEM containing cycloheximide (20 μg/ml) and incubated at 32˚C for 30 min. Staining was performed with primary antibodies, mouse anti-GM130 (1:250) (Golgi marker) (BD Transduction) and rabbit anti-calnexin (1:250) (ER marker) (Abcam) overnight. Secondary antibodies AlexaFluor 647 goat anti-mouse (1:200) and goat anti-rabbit (1:200) (Invitrogen) were used. Mander's coefficient (M) was used to determine the degree of co-localisation between VSVG^{ts045}-mCherry and the ER or Golgi marker. Mander's coefficient was calculated for twenty cells by JACoP (Colocalisation Plugin) (Bolte and Cordelieres, 2006) in ImageJ (NIH). Twenty cells were scored in each experiment and all experiments were performed in triplicate. Plugins were used in ImageJ, and the measuring areas were selected above a threshold against background staining. After analysis, Mander's coefficient in the range from 0 to 1.0 (representing 0–100% overlapping pixels) was calculated to determine overlap between images.

Treatment with BMC and BSO

Treatment with trans-1,2-bis mercaptoacetamido cyclohexane (BMC) (Toronto-ResearchChemicals) was added from a stock dissolved at 25 µM in DMSO. BMC was added to the transfected cells expressing SOD1, TDP-43, 4 hr post transfection. 75 µM BSO was administered into transfected cells 24 hr post transfection for a total of 48 hr. Cells were fixed 72 hr post transfection and an immunofluorescence assay was performed. For FACS, 6 hr after transfection, the culture medium was replaced by fresh medium added with 25 µM BMC or 75 µM BSO and the incubation extended for 16 hr.

Flow cytometry assays

Neuro-2a cells were transfected with 0.4 µg of vector encoding the fluorescent redox biosensor Clover-rxmRuby2 (RXM) (Piattoni et al., 2019) or co-transfected with 1.2 µg of pCDNA3.1 encoding PDI (DNA ratio RXM and PDI = 1:3), either PDI-WT, PDI-QUAD, PDI-D292N or PDI-R300H. Neuro-2a transfected cells were trypsinized and resuspended in fresh culture medium before flow cytometry analysis with a BD FACSAria™ Fusion. For each sample, 30,000 counts gated on an FSC vs. SSC dot plot excluding cell doublets were recorded. The settings used for acquisition of fluorescence signals were λex/λem = 488/ 530 ± 15 nm for Clover and λex/λem = 561/610 ± 10 nm for rxmRuby2. Quantitative assessment was performed for Clover and rxmRuby2 double positive cells. The intracellular redox state of rxmRuby2 was achieved by normalizing the mean fluorescence intensity (FI) of rxmRuby2 to Clover, mean FI and by calibrating the measured signal to maximum and minimum FI values of both fluorescent proteins obtained from cells exposed to redox stimuli yielding reduced (5 mM DTT 60 min) and oxidized (100 µM diamide 30 min) redox biosensor, respectively. The programs BD FACSDIVA V8.0.1 and FlowJo v.7.6.5 (BD Biosciences) were used for data analysis, n=2.

Generation of mRNA for microinjection into zebrafish embryos

Human SOD1 mRNA was generated as described previously (Robinson et al., 2018). Firstly, a pCMV construct containing human wild-type or mutant (A4V) SOD1 DNA was linearized by PciI restriction enzyme digest, followed by purification using QIAquick Gel Extraction Kit (Qiagen). 1 µg of purified DNA was then transcribed using a T7 mMESSAGE mMACHINE In Vitro Transcription kit (Ambion, Applied Bioscience), followed by purification via MEGAclear transcription clean-up kit (Ambion, Applied Bioscience). To generate mRNA encoding human PDI (wild type or the QUAD mutant) the pCS2P and PDI-mKate2 construct was firstly linearized with NotI and the DNA purified. *In vitro* transcription was

then performed using a SP6 mMESSAGE mMACHINE In Vitro Transcription kit (Ambion, Thermofisher, catalogue AM1340,) followed by purification using a MEGAclear transcription clean-up kit (Ambion, Applied Bioscience).

Zebrafish maintenance and mRNA microinjection

All animal husbandry and experimental procedures were performed in compliance with the Animal Ethics Committee, Macquarie University, NSW, Australia (ARA 2015/034 and ARA 2017/019) and the Internal Biosafety Committee, Macquarie University (NLRD 5201401007). Transgenic zebrafish expressing blue fluorescent protein in their motor neurons on a TAB_WT background Tg (-3mnx1:TagBFP)mq10[28] were bred and maintained under established conditions. The adult zebrafish were mated and the resulting embryos collected for microinjection experiments. Human SOD1 mRNA (250 ng/µl) was co-injected, along with either mKate2 mRNA or mRNA encoding PDI (WT or QUAD) fused to mKate2 (200 ng/µl) depending on experimental group, *via* a 0.905 nl droplet into 1-4 cell stage zebrafish embryos using a Picospritzer II (Parker Instrumentation). At 24 hpf embryos were screened for successful injection (expression of mKate2 red fluorescent protein) using a M165FC fluorescent stereomicroscope (Leica) and positive embryos were manually dechorionated with forceps and raised in equal numbers in darkness at 28°C. At 48hpf morphologically normal larvae were distributed into a 96-well plate, with each embryo placed within a separate well containing 250 μl of E3 medium (5 nM NaCl, 0.17 mM KCl, 0.33 mM $CaCl_2$ and 0.33 mM $MgSO_4$,). The micro-well plate was then incubated at 28°C for 15 min prior to launching the movement tracking assay.

BMC treatment of zebrafish embryos

Treatment of mutant SOD1 expressing zebrafish with BMC was performed through addition of the compound to the E3 incubation media. At 24 hpf embryos positive for red fluorescence were manually de-chorionated and distributed into small petri dishes containing either 12.5µM BMC in E3 solution or DMSO in E3 as a control. The embryos were then incubated in darkness at 28°C until they were transferred to 96-well plates (one per well) for motor tracking at 48 hpf.

Tracking movement of zebrafish embryos

Only morphologically normal embryos were used in the movement tracking analysis, which was performed using a Zebrabox (Viewpoint) automated zebrafish movement recording device as described

previously (Robinson et al., 2018). At 48 hpf, 96-well plates containing the zebrafish larvae were placed inside the Zebrabox device and left for 30 min to allow the zebrafish larvae to acclimatise to the dark conditions. A test of the motor response to a flash of light was then performed, involving exposing the animals to a 1 second flash of 300 watts of light. This light stimulus was repeated three times at 1 min intervals. The total distance travelled by each animal during the 3 min test period (starting from first flash of light) was recorded.

Statistics

Statistical comparisons between group means were performed using GraphPad Prism 6 software (Graph Pad software, Inc.). For the zebrafish behavioural studies, the data relating to the total distance swum did not meet assumptions to allow ANOVA analysis, so comparisons were made using a Kruskal-Wallis test followed by *post hoc* Dunn's test for multiple comparisons. ***p<0.0001, **p=0.004, *p=0.021. Results were calculated as standard error of the mean (SEM). For cell culture experiments, 100 transfected cells were examined unless specified in the figure legend and 35 primary neurons were examined in each group in each experiment. Results were expressed as mean ± SD, n = 3, unless specified otherwise. Statistical analyses were made using ANOVA followed by Tukey's *post-hoc* test (GraphPad Prism 6, San Diego, CA). *p*-values of 0.05 or less were considered significant: *p<0.05, **p<0.01, ***p<0.001, ****p<0.0001.

Supplementary Results

S1A

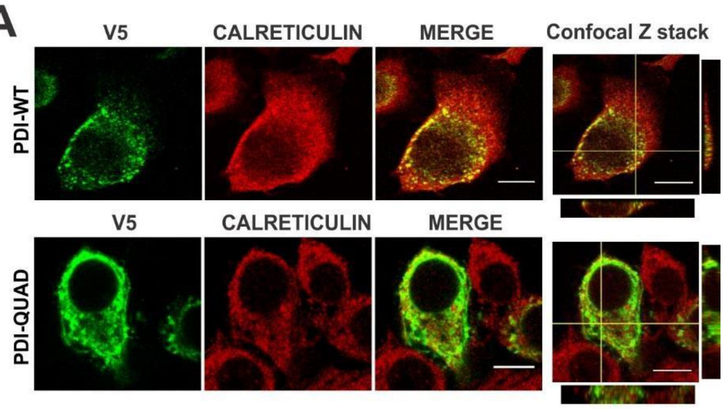

S1B

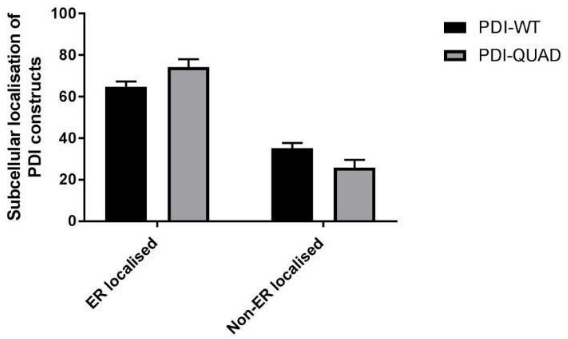

S1) Expression of PDI in Neuro-2a cells, related to Figure 1A.

S1A) Confocal microscopy images of wild-type PDI (PDI-WT) and mutant PDI (PDI-QUAD) expressed in Neuro-2a cells, 24 hr post-transfection. Cells were probed with an anti-V5-antibody to stain PDI (first column) and anti-calreticulin (second column) antibodies using immunocytochemistry. Merge of the fluorescent images obtained from immunocytochemistry using anti-V5 and anti-calreticulin antibodies is shown in the third column. Orthogonal section of the confocal images (fourth column) showing co-localization of PDI-WT and PDI-QUAD with ER marker calreticulin. Scale bar = 12 μm. **S1B)** Quantification of the degree of co-localization of PDI with calreticulin (S1A) using Mander's coefficient demonstrated that 65% of PDI-WT colocalised with calreticulin, while 35% was observed in a non-ER localisation. Similarly, 75% of PDI-QUAD co-localised with calreticulin and 25% was observed in a non-ER location.

S2A

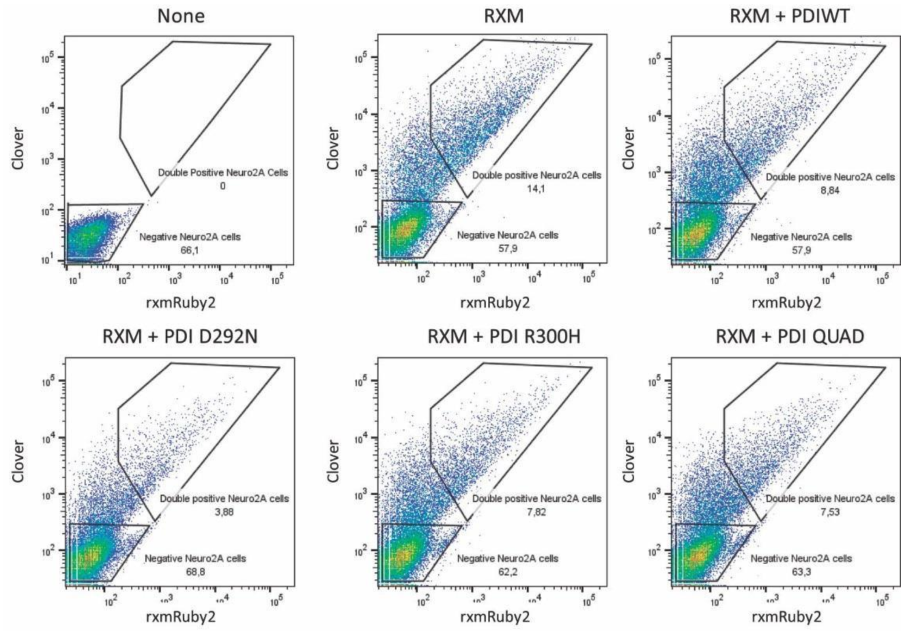

S2) Flow cytometry analysis of Neuro-2A cells expressing PDI-WT or mutants QUAD, D292N or R300H and the redox biosensor Clover-rxmRuby2*, related to Figure 1A.*

S2A) Representative dot plots of Clover (λex/λem = 488/ 530 ± 15 nm) vs. rxmRuby2 (λex/λem = 561/610 ± 10 nm) fluorescence for untransfected Neuro-2a cells (None) or transiently transfected with the Clover-rxmRuby2 biosensor (RXM) or, in addition, with either PDI: PDI-WT, PDI-QUAD, PDI-D292N or PDI-R300H. The small (bottom left corner) and the large gate (upper right position) denotes the population of Clover/rxmRuby2 negative and double positive cells, respectively. The inset values indicate the corresponding percentages of double negative or double positive fluorescent cells.

S3A

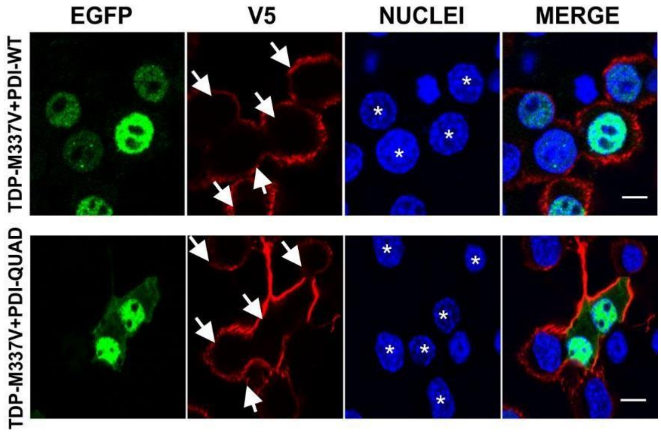

S3) Co-expression of TDP-43 and PDI in Neuro-2a cells, related to Figure 2A.

S3A) Representative confocal microscopy images of cells examined at 72 hr post transfection co-expressing TDP-M337V (green) and PDI- WT (red) with Hoechst-stained nuclei (blue) (panel 1), and co-expressing TDP-M337V (green) and PDI-QUAD (red) with Hoechst-stained nuclei (blue) (panel 2). The fourth column is a merge of the fluorescent images of GFP and V5. Scale bar = 10 μm.

S4A

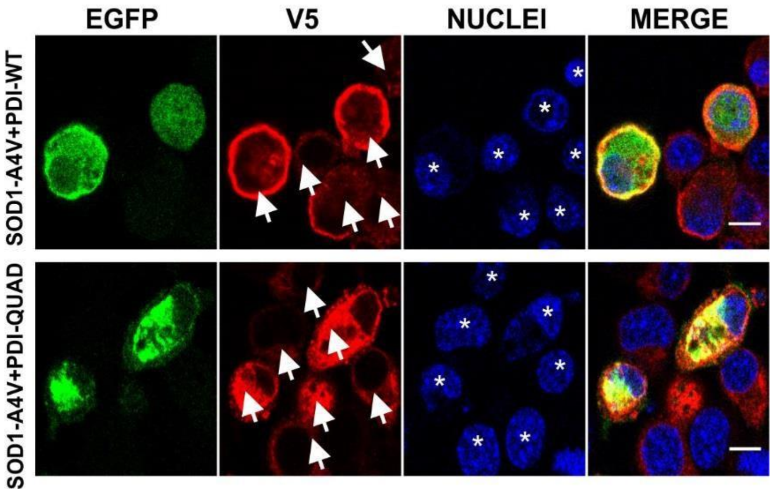

S4) Co-expression of SOD1 and PDI in Neuro-2a cells, related to Figure 4A.

S4A) Representative confocal microscopy images of cells examined 72 hr post transfection co-expressing SOD1-A4V (green) and PDI-WT (red) with Hoechst-stained nuclei (blue) (panel 1), and co-expressing SOD1-A4V (green) and PDI-QUAD (red) with Hoechst-stained nuclei (blue) (panel 2). The fourth column is a merge of the fluorescent images of GFP and V5. Scale bar = 10 μm.

S5A

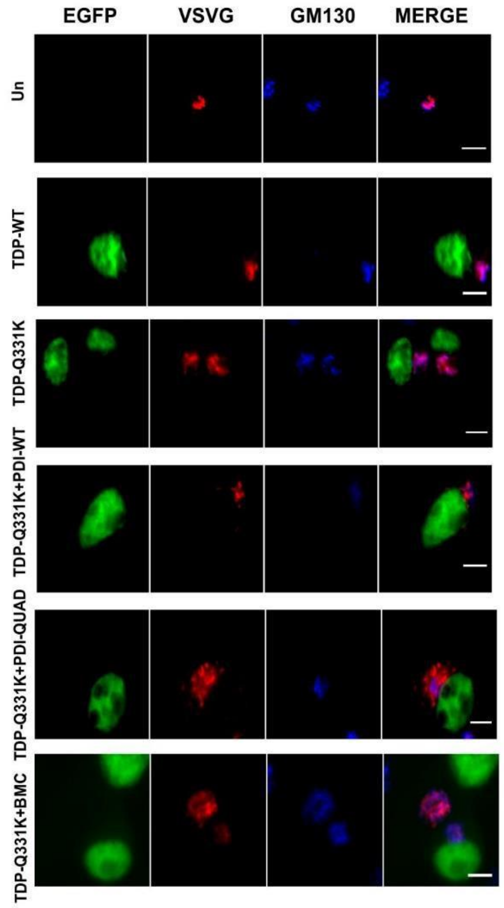
EGFP
VSVG
GM130
MERGE
Un
TDP-WT
TDP-Q331K
TDP-Q331K+PDI-WT
TDP-Q331K+PDI-QUAD
TDP-Q331K+BMC

S5B

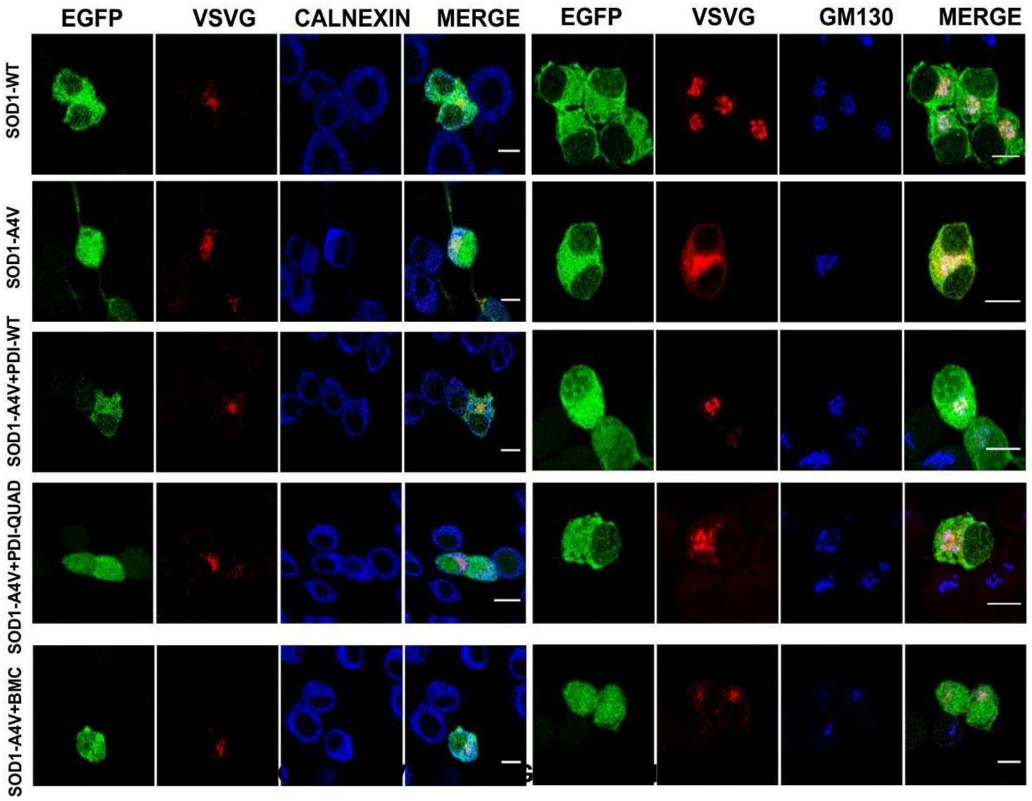
EGFP
VSVG
CALNEXIN
MERGE
EGFP
VSVG
GM130
MERGE
SOD1-WT
SOD1-A4V
SOD1-A4V+PDI-WT
SOD1-A4V+PDI-QUAD
SOD1-A4V+BMC

S5) ***The oxidoreductase activity of PDI is protective against ER-Golgi transport defects induced by mutant TDP-43 and mutant SOD1.***

S5A) Representative fluorescent images of cells co-expressing VSVGts045-mCherry with EGFP-tagged TDP-WT or TDP-Q331K, and PDI (WT or QUAD) at 16 hr post-transfection, stained with markers of Golgi apparatus (GM130, related to Figure 5G). **S5B)** Representative images of cells co- expressing VSVG^{ts045}-mCherry, SOD1-WT (row 1) or SOD1-A4V VSVG^{ts045}-mCherry (row 2), or PDI- WT, or PDI-QUAD, or treated with BMC (row 3, 4, 5) stained with the markers of ER (calnexin) and Golgi apparatus (GM130, related to Figure 6E).

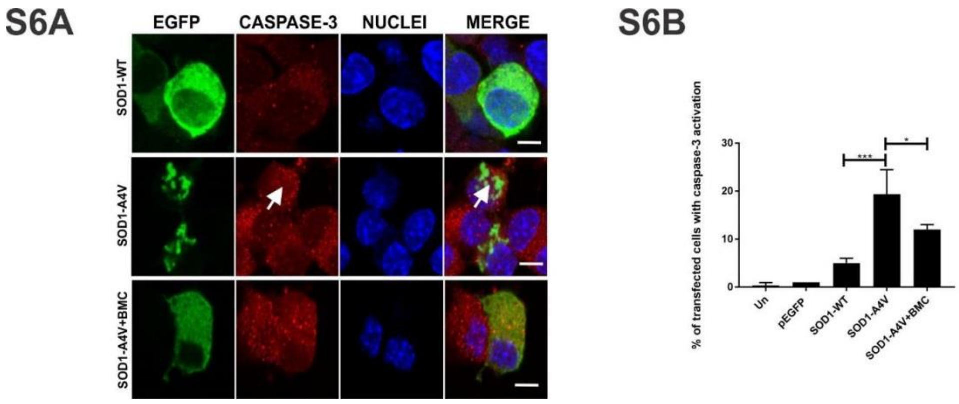

S6) BMC is protective against mutant SOD1 induced cell death, related to Figure 8C.

S6A) Immunocytochemistry using anti-activated caspase-3 antibodies (red), white arrow represents caspase-3 activation, indicating apoptosis is underway. As expected, fewer cells expressing SOD1-WT (row 1) displayed caspase-3 activation, compared to cells expressing SOD1-A4V (row 2). However, fewer cells treated with BMC in SOD1-A4V expressing populations (row 3) displayed caspase-3 activation, compared to those SOD1-A4V cells transfected with empty vector. Scale bar = 12 µm **S6B)** Quantification of caspase-3 positive cells per group visualized in (S6A). Treatment with BMC (*p<0.05) in SOD1-A4V expressing cells significantly decreased the proportion of cells with activated caspase-3.

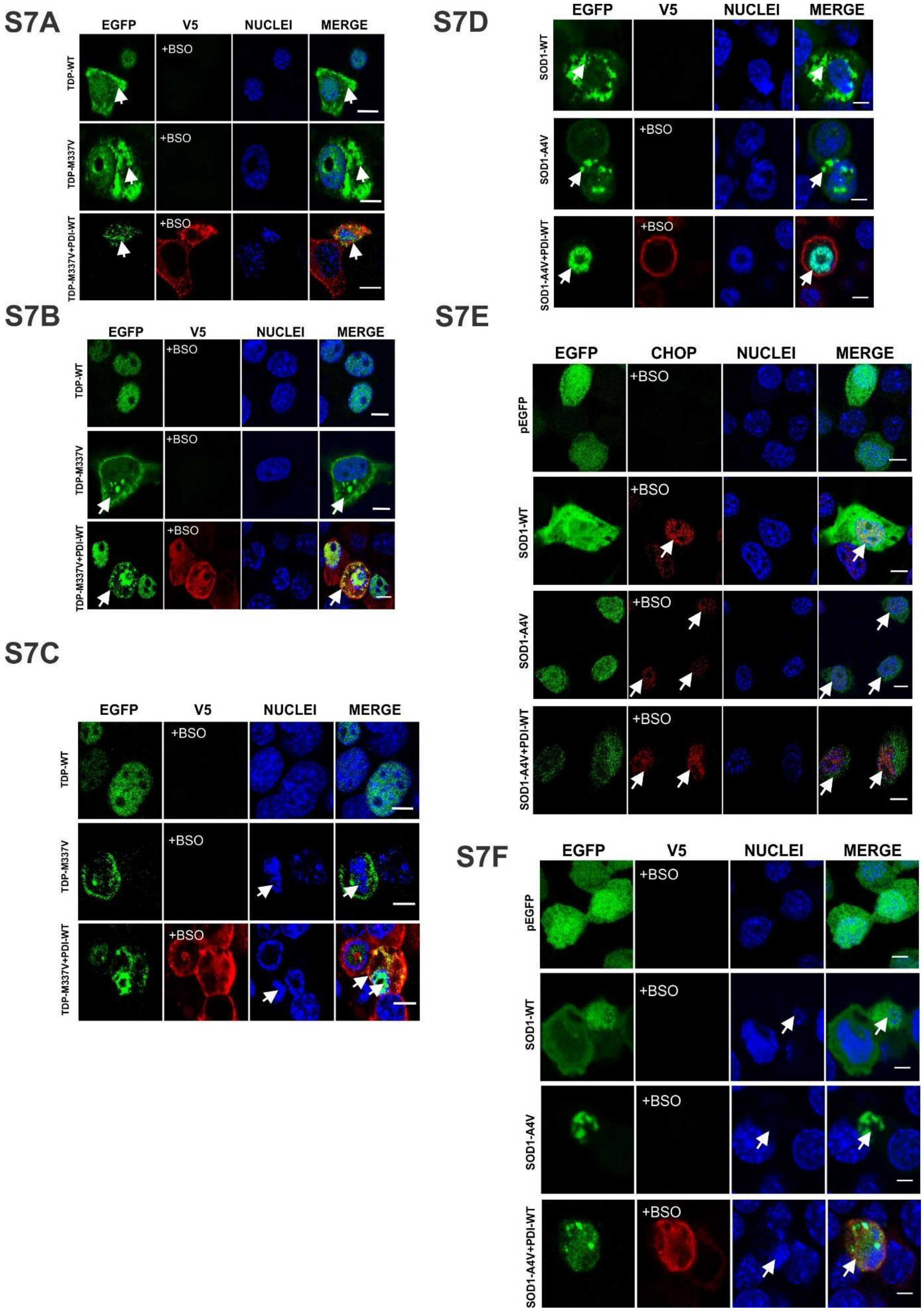
S7A
EGFP
V5
NUCLEI
MERGE
TDP-WT
TDP-M337V
TDP-M337V+PDI-WT
+BSO
S7B
EGFP
V5
NUCLEI
MERGE
TDP-WT
TDP-M337V
TDP-M337V+PDI-WT
+BSO
S7C
EGFP
V5
NUCLEI
MERGE
TDP-WT
TDP-M337V
TDP-M337V+PDI-WT
+BSO
S7D
EGFP
V5
NUCLEI
MERGE
SOD1-WT
SOD1-A4V
SOD1-A4V+PDI-WT
+BSO
S7E
EGFP
CHOP
NUCLEI
MERGE
pEGFP
SOD1-WT
SOD1-A4V
SOD1-A4V+PDI-WT
+BSO
S7F
EGFP
V5
NUCLEI
MERGE
pEGFP
SOD1-WT
SOD1-A4V
SOD1-A4V+PDI-WT
+BSO

S7) Depletion of intracellular GSH by biochemical inhibition using BSO abrogates protective activity of PDI against mutant TDP-43 and mutant SOD1*, related to Figure 9*.

S7A) Inclusions were observed in cells expressing TDP-WT and TDP-M337V upon treatment with BSO compared to DMSO (row 1 and 2). Moreover, significantly more cells co-expressing PDI-WT with TDP-M337V (row 3) formed inclusions following BSO treatment compared to cells co-expressing PDI-WT and TDP-M337V with DMSO treatment. Scale bar = 10 µm. **S7B)** Neuro-2a cells expressing TDP-WT (row 1) displayed similar cytoplasmic TDP-43 localisation following BSO treatment compared to DMSO treatment. Cells expressing TDP-M337V exhibited more cytoplasmic localisation (row 2) upon BSO treatment. BSO treatment also led to more cells with cytoplasmic TDP-43 distribution in PDI-WT with TDP-M337V populations (row 3). Scale bar = 8 µm. **S7C)** Neuro-2a cells expressing TDP-WT (row 1) contained less fragmented nuclei when treated with BSO. Cells expressing TDP-M337V with or without BSO treatment (row 2) displayed apoptotic nuclei. More cells co-expressing TDP-M337V with PDI-WT upon BSO administration were undergoing apoptosis compared to DMSO treatment (row 3). Scale bar = 10 µm. **S7D)** Immunofluorescence detection of EGFP tagged SOD1 in Neuro-2a cells co-expressed with V5-taged PDI-WT and treated with BSO or DMSO. Inclusions were observed in cells expressing SOD1-WT upon treatment with BSO (row 1) unlike those treated with vehicle DMSO. Inclusion formation was similar in cells expressing mutant SOD1-A4V with empty vector (row 2) with or without BSO. More cells co-expressing PDI-WT with SOD1-A4V (row 3) formed inclusions upon BSO treatment compared to those treated with DMSO. Scale bar = 5 µm. **S7E)** Neuro-2a cells expressing EGFP only treated with/without BSO (row 1) displayed little nuclear CHOP activation. More CHOP activation was observed in cells expressing SOD1-WT upon treatment with BSO compared to untreated cells (row 2). CHOP activation was similar in cells expressing mutant SOD1-A4V with empty vector in samples treated with (row 3) or without BSO. More cells co-expressing PDI-WT with SOD1-A4V (row 4) displayed CHOP activation upon BSO treatment compared to those treated with DMSO. Scale bar = 5 µm. **S7F)** Few cells expressing EGFP (row 1) contained fragmented nuclei. Apoptotic nuclei were detected in cells expressing SOD1-WT upon treatment with BSO but not with DMSO (**$p<0.01$, row 2). Cells expressing SOD1-A4V with or without BSO treatment (row 3) displayed fragmented nuclei. More cells co-expressing SOD1-A4V with PDI-WT upon BSO administration (row 4) were apoptotic. Scale bar = 5 µm.

Supplemental References

BOLTE, S. & CORDELIERES, F. 2006. A guided tour into subcellular colocalization analysis in light microscopy. *Journal of microscopy,* 224**,** 213-232.

FARRAWELL, N. E., LAMBERT-SMITH, I. A., WARRAICH, S. T., BLAIR, I. P., SAUNDERS, D. N., HATTERS, D. M. & YERBURY, J. J. 2015. Distinct partitioning of ALS associated TDP-43, FUS and SOD1 mutants into cellular inclusions. *Scientific Reports,* 5**,** 1-14.

JESSOP, C. E., WATKINS, R. H., SIMMONS, J. J., TASAB, M. & BULLEID, N. J. 2009. Protein disulphide isomerase family members show distinct substrate specificity: P5 is targeted to BiP client proteins. *Journal of cell science,* 122**,** 4287-4295.

PARAKH, S., JAGARAJ, C. J., VIDAL, M., RAGAGNIN, A. M. G., PERRI, E. R., KONOPKA, A., TOTH, R. P., GALPER, J., BLAIR, I. P., THOMAS, C. J., WALKER, A. K., YANG, S., SPENCER, D. M. & ATKIN, J. D. 2018. ERp57 is protective against mutant SOD1-induced cellular pathology in amyotrophic lateral sclerosis. *Human Molecular Genetics,* 27**,** 1311-1331.

PIATTONI, C. V., SARDI, F., KLEIN, F., PANTANO, S., BOLLATI-FOGOLIN, M. & COMINI, M. 2019. New red-shifted fluorescent biosensor for monitoring intracellular redox changes. *Free Radical Biology and Medicine,* 134**,** 545-554.

ROBINSON, K. J., YUAN, K. C., DON, E. K., HOGAN, A. L., WINNICK, C. G., TYM, M. C., LUCAS, C. W., SHAHHEYDARI, H., WATCHON, M. & BLAIR, I. P. 2018. Motor Neuron Abnormalities Correlate with Impaired Movement in Zebrafish that Express Mutant Superoxide Dismutase 1. *Zebrafish*.

TURNER, B. J., ATKIN, J. D., FARG, M. A., ZANG, D. W., REMBACH, A., LOPES, E. C., PATCH, J. D., HILL, A. F. & CHEEMA, S. S. 2005. Impaired extracellular secretion of mutant superoxide dismutase 1 associates with neurotoxicity in familial amyotrophic lateral sclerosis. *Journal of Neuroscience,* 25**,** 108-117.

WALKER, A. K., SOO, K. Y., SUNDARAMOORTHY, V., PARAKH, S., MA, Y., FARG, M. A., WALLACE, R. H., CROUCH, P. J., TURNER, B. J., HORNE, M. K. & ATKIN, J.D. 2013. ALS-associated TDP-43 induces endoplasmic reticulum stress, which drives cytoplasmic TDP-43 accumulation and stress granule formation. *PloS one,* 8**,** e81170.

WOEHLBIER, U., COLOMBO, A., SAARANEN, M. J., PÉREZ, V., OJEDA, J., BUSTOS, F. J., ANDREU, C. I., TORRES, M., VALENZUELA, V. & MEDINAS, D. B. 2016. ALS-linked protein disulfide isomerase variants cause motor dysfunction. *The EMBO journal,* 35**,** 845-865.
